# Supplementary material for: Systematic Evaluation of Normalization Methods for Glycomics Data Based on Performance of Network Inference
Source: Metabolites. 2020 Jul 2;10(7):271. doi: 10.3390/metabo10070271 (PMC7408386; doi:10.3390/metabo10070271)
Supplement: Supplementary file 1 [file metabolites-10-00271-s001.zip › SupplementaryFigures.pdf]

Supplementary Figures

Figure S1: Reference pathway for IgG UHPLC-FLD data. IgG glycans include monosaccharides such as mannose, N-acetylglucosamine, galactose, fucose and sialic acid. Since each of this platform measures a slightly different set of IgG glycans than LC-ESI-MS, the reference pathway was adapted to fit the structures quantified in the respective dataset.

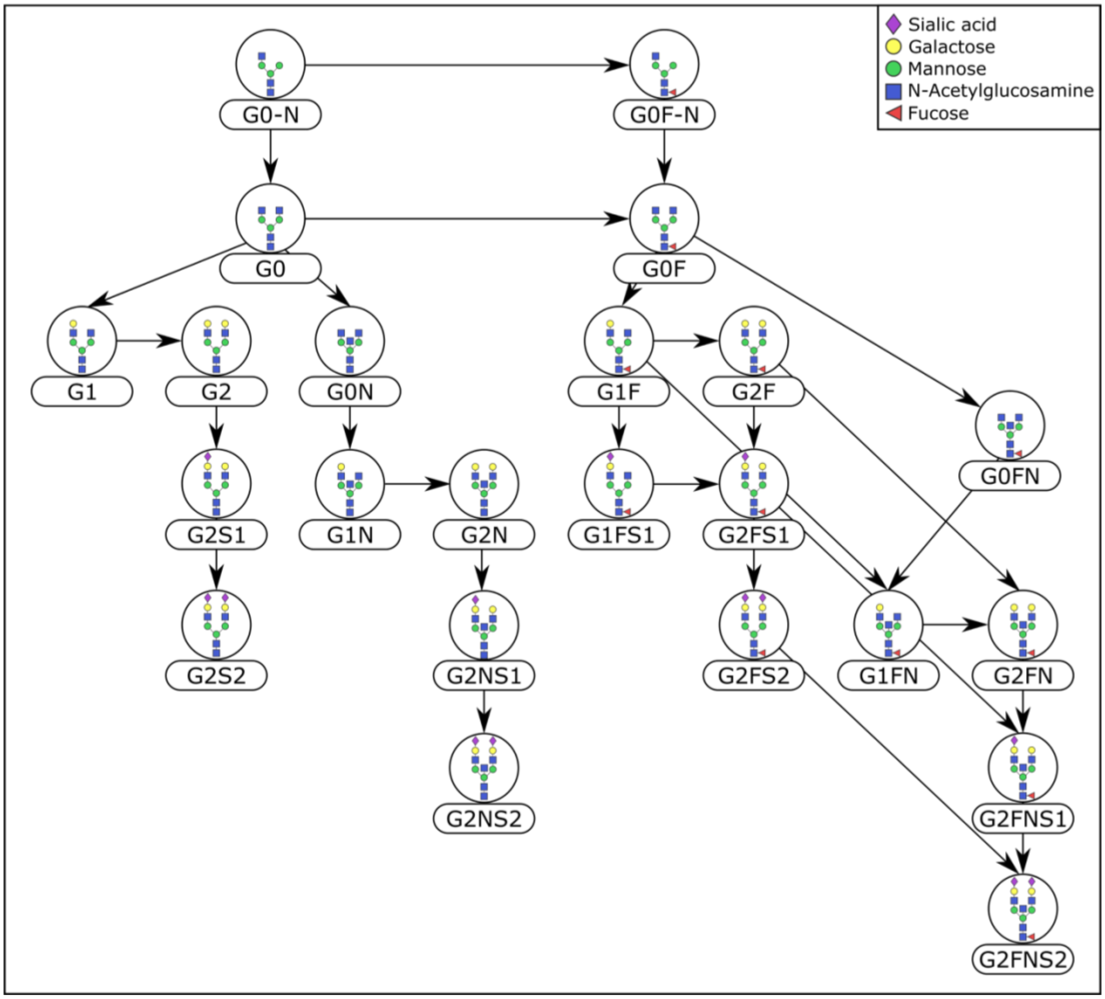

Figure S2: Hypothetical pathway of total plasma N-glycan synthesis (see next page). Each node corresponds to a glycan structure. Edges represent possible enzymatic reactions of glycan synthesis, namely all single monosaccharide modifications against which there is no strong experimental evidence. **A** In each node, the corresponding glycan structure is depicted. **B** Each node is labelled with the composition of the corresponding structure. White nodes indicate that the corresponding composition was not measured in the dataset. Gray nodes indicate compositions that can be attributed to a single glycan structure. Colored nodes indicate that there are multiple structures with the same composition.

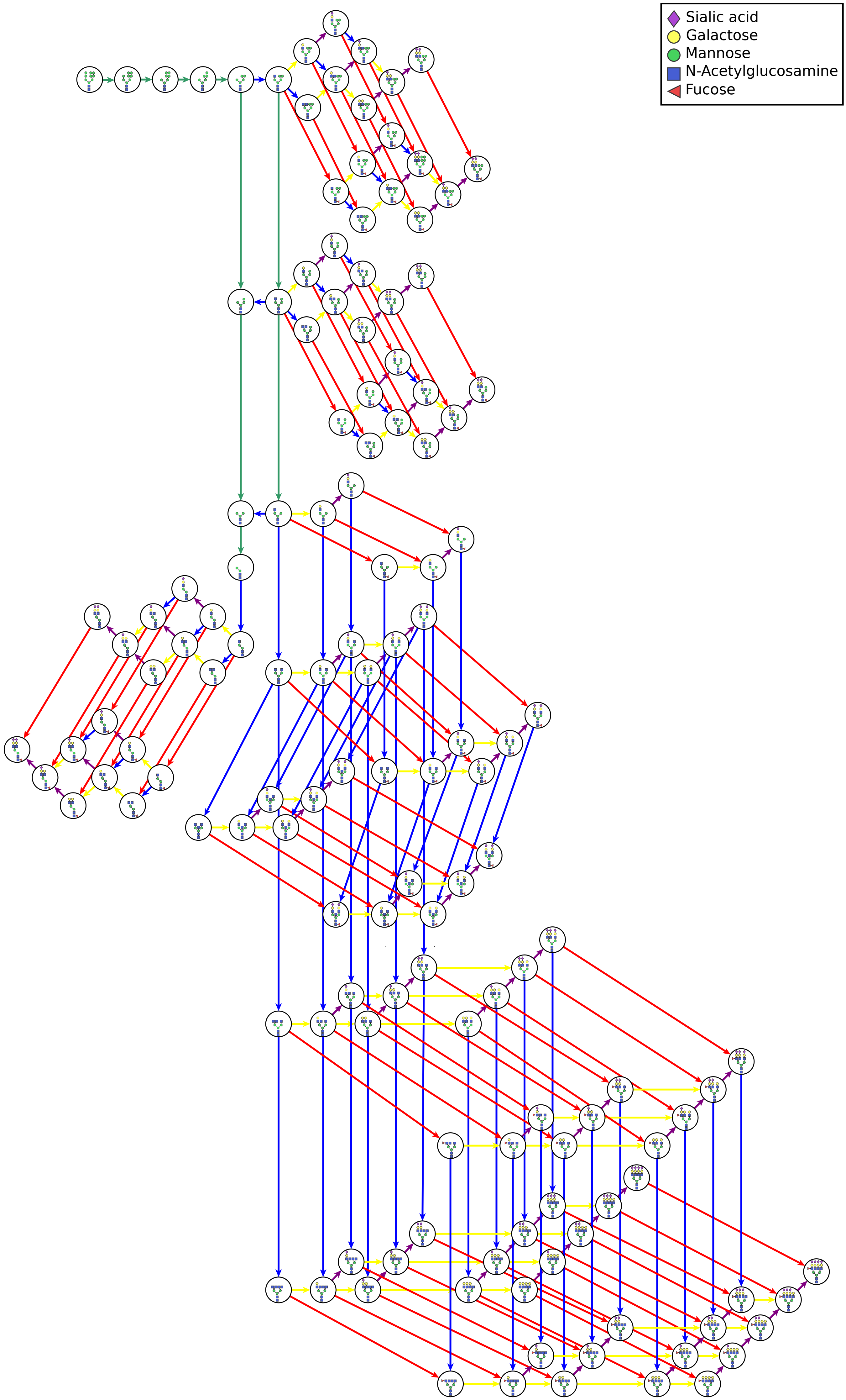
Sialic acid
Galactose
Mannose
N-Acetylglucosamine
Fucose

B

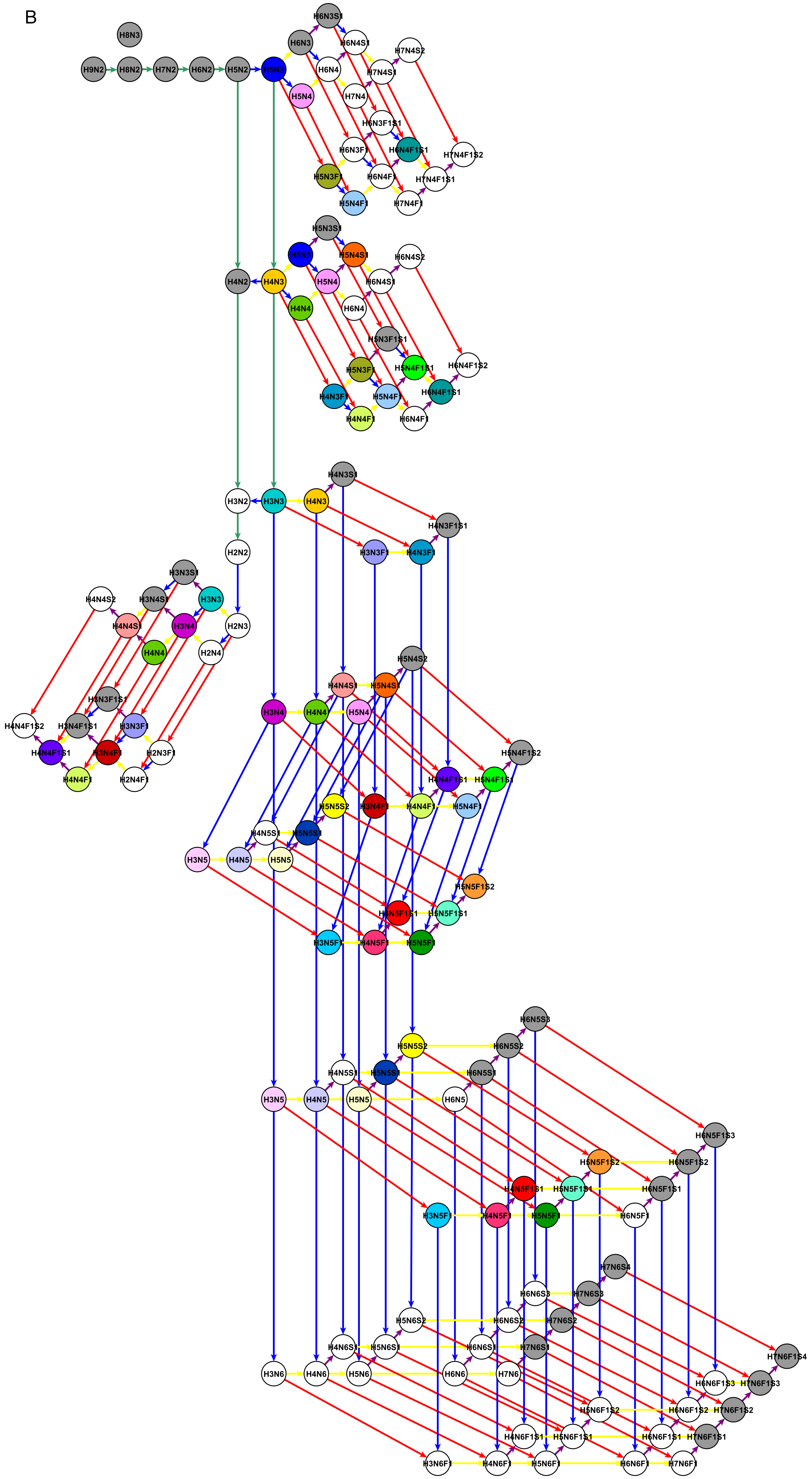

H6N3S1
H8N3
H6N3
H6N4S1
H7N4S2
H9N2
H8N2
H7N2
H6N2
H5N2
H5N3
H6N4
H7N4S1
H5N4
H7N4
H6N3F1S1
H6N3F1
H6N4F1S1
H7N4F1S2
H5N3F1
H6N4F1
H7N4F1S1
H5N4F1
H7N4F1
H5N3S1
H5N3
H5N4S1
H6N4S2
H4N2
H4N3
H5N4
H6N4S1
H4N4
H6N4
H5N3F1S1
H5N3F1
H5N4F1S1
H6N4F1S2
H4N3F1
H5N4F1
H6N4F1S1
H4N4F1
H6N4F1
H4N3S1
H3N2
H3N3
H4N3
H4N3F1S1
H2N2
H3N3F1
H4N3F1
H3N3S1
H4N4S2
H3N4S1
H3N3
H4N4S1
H3N4
H2N3
H4N4
H2N4
H5N4S2
H3N3F1S1
H4N4S1
H5N4S1
H4N4F1S2
H3N4F1S1
H3N3F1
H3N4
H4N4
H5N4
H5N4F1S2
H4N4F1S1
H3N4F1
H2N3F1
H4N4F1S1
H5N4F1S1
H4N4F1
H2N4F1
H5N5S2
H3N4F1
H4N4F1
H5N4F1
H4N5S1
H5N5S1
H3N5
H4N5
H5N5
H5N5F1S2
H4N5F1S1
H5N5F1S1
H3N5F1
H4N5F1
H5N5F1
H6N5S3
H5N5S2
H6N5S2
H4N5S1
H5N5S1
H6N5S1
H3N5
H4N5
H5N5
H6N5
H6N5F1S3
H5N5F1S2
H6N5F1S2
H4N5F1S1
H5N5F1S1
H6N5F1S1
H3N5F1
H4N5F1
H5N5F1
H6N5F1
H7N6S4
H6N6S3
H7N6S3
H5N6S2
H6N6S2
H7N6S2
H4N6S1
H5N6S1
H6N6S1
H7N6S1
H7N6F1S4
H3N6
H4N6
H5N6
H6N6
H7N6
H6N6F1S3
H7N6F1S3
H5N6F1S2
H6N6F1S2
H7N6F1S2
H4N6F1S1
H5N6F1S1
H6N6F1S1
H7N6F1S1
H3N6F1
H4N6F1
H5N6F1
H6N6F1
H7N6F1

Figure S3: Creation of the reference pathway for the MALDI-FTICR-MS data. Starting from the hypothetical biochemical pathway (**A**) and the mapping between structures in the hypothetical pathway and the corresponding compositional name (**B**), structures with the same mass in the canonical pathway were merged into one single compositional node (**C**). Since not all compositions in the canonical pathway were measured in the available dataset, the unmeasured compositions were removed from the pathway together with all their edges (**D**).

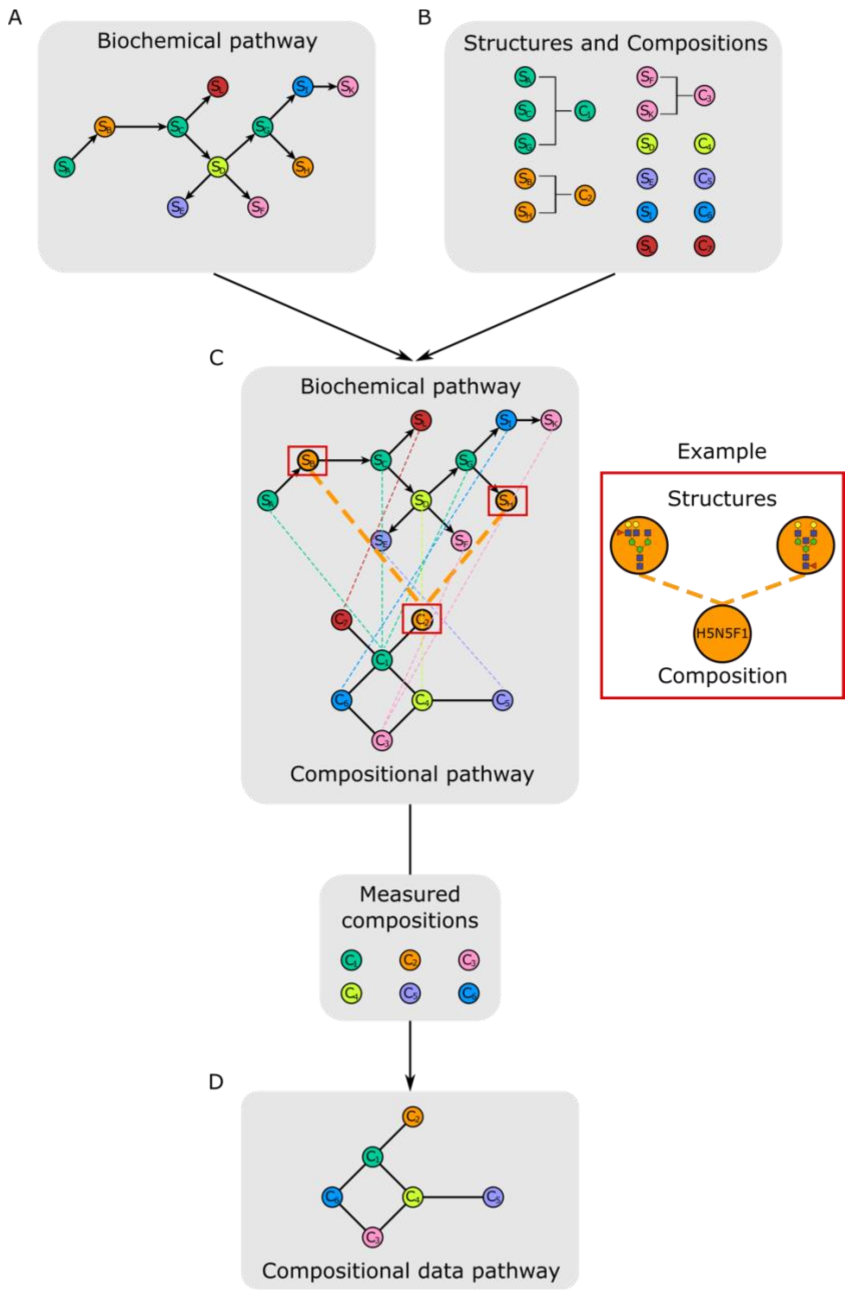

Figure S4: Reference pathway for total plasma N-glycans in MALDI-FTICR-MS data. This technology separates glycans based on their total mass, which can then be characterized according to the contained number of hexoses (like glucose, galactose and mannose, indicated with H), N-acetylhexosamines (N-acetylglucosamine, indicated with N), fucoses (F), and sialic acids (S). Grey nodes represent compositions made of a single structure, while colored nodes represent compositions made of more than one structure, as indicated in Figure S2B.

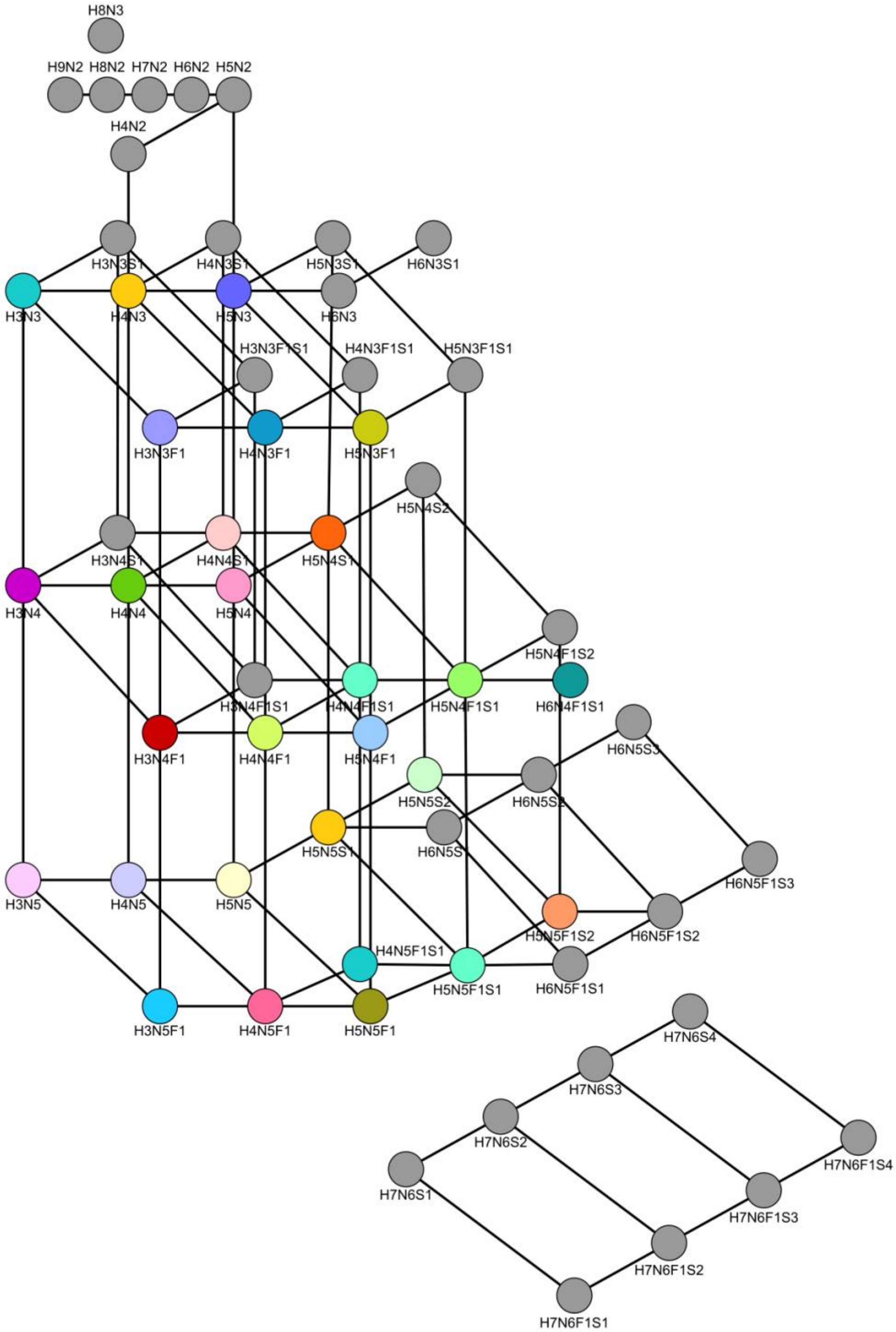

Figure S5: LC-ESI-MS normalization analysis results (Korčula 2010 cohort). Results in the panels are colored according to type of normalization (left), log-transformation (center), or normalization per IgG subclass or total IgG (right). Bars represent the median of the Fisher's exact test p-values over 1,000 bootstrapping, and error bars the corresponding 95% confidence intervals.

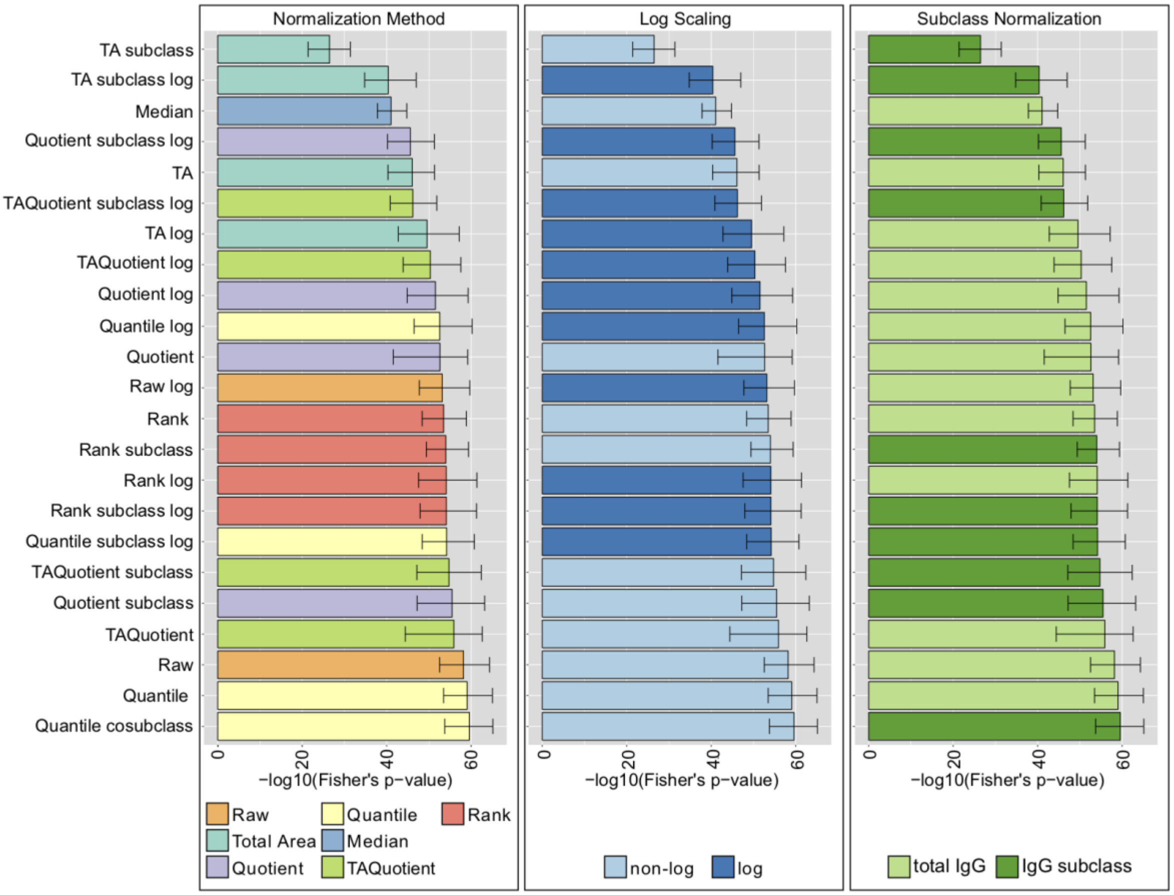

Figure S6: LC-ESI-MS normalization analysis results (Split cohort). Results in the panels are colored according to type of normalization (left), log-transformation (center), or normalization per IgG subclass or total IgG (right). Bars represent the median of the Fisher's exact test p-values over 1,000 bootstrapping, and error bars the corresponding 95% confidence intervals.

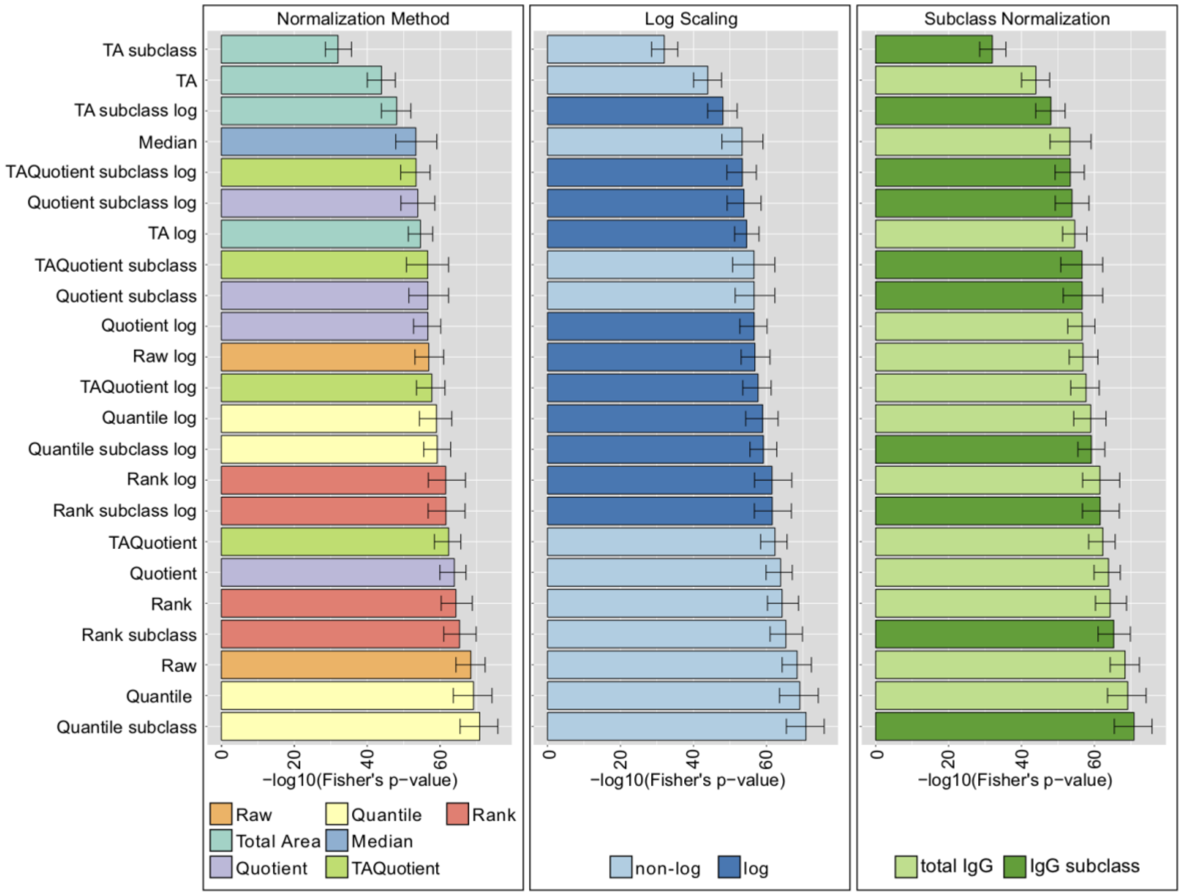

Figure S7: LC-ESI-MS normalization analysis results (Vis cohort). Results in the panels are colored according to type of normalization (left), log-transformation (center), or normalization per IgG subclass or total IgG (right). Bars represent the median of the Fisher's exact test p-values over 1,000 bootstrapping, and error bars the corresponding 95% confidence intervals.

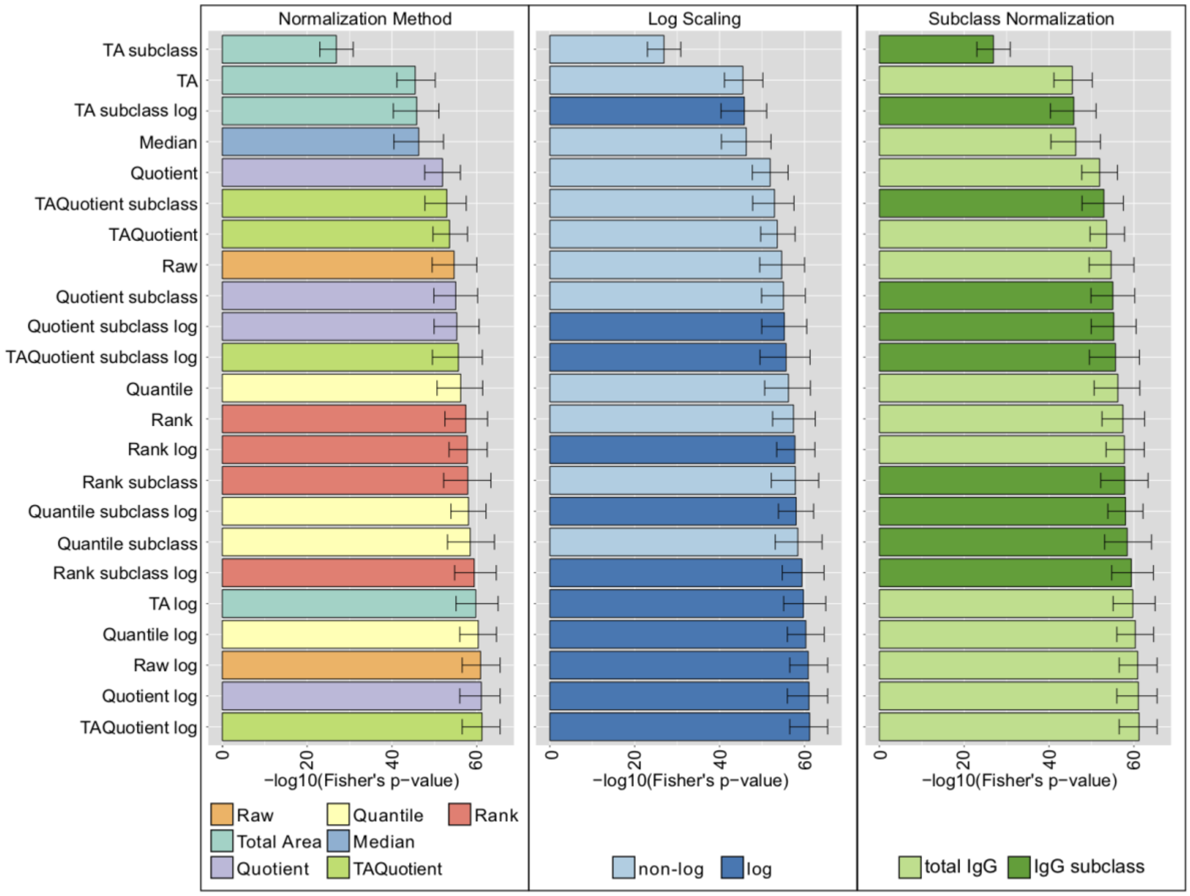

Figure S8: associations with age (see next page). Each panel displays the adjusted p-value of the association of the corresponding glycan feature with age for the different normalizations considered in the six cohorts considered. The black dashed line represents the False Discovery Rate 0.05 significance threshold. Therefore, all points above the line indicate a statistically significant association.

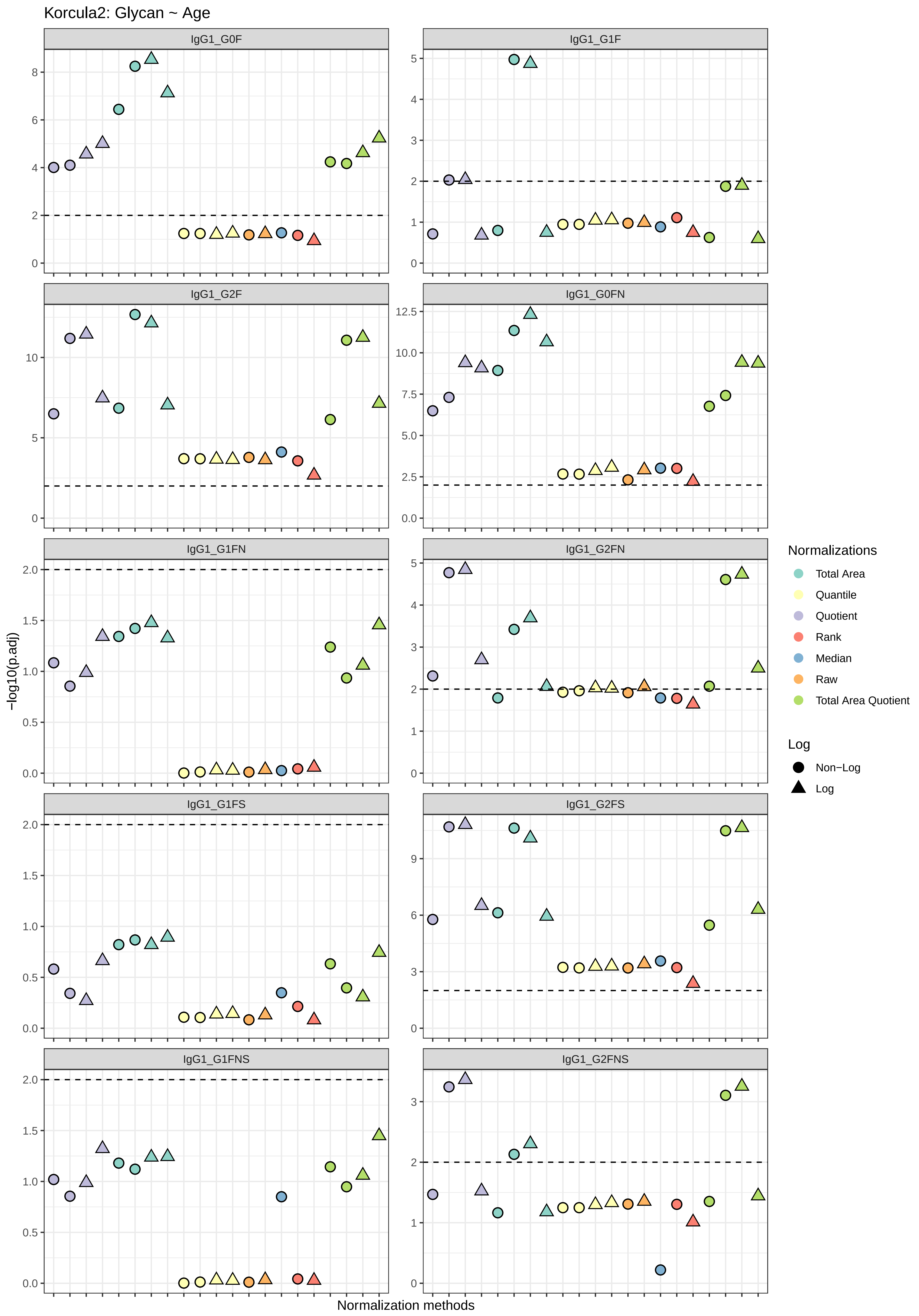

Korcula2: Glycan ~ Age
IgG1_G0F
IgG1_G1F
IgG1_G2F
IgG1_G0FN
IgG1_G1FN
IgG1_G2FN
IgG1_G1FS
IgG1_G2FS
IgG1_G1FNS
IgG1_G2FNS
−log10(p.adj)
Normalization methods
Normalizations
Total Area
Quantile
Quotient
Rank
Median
Raw
Total Area Quotient
Log
Non−Log
Log

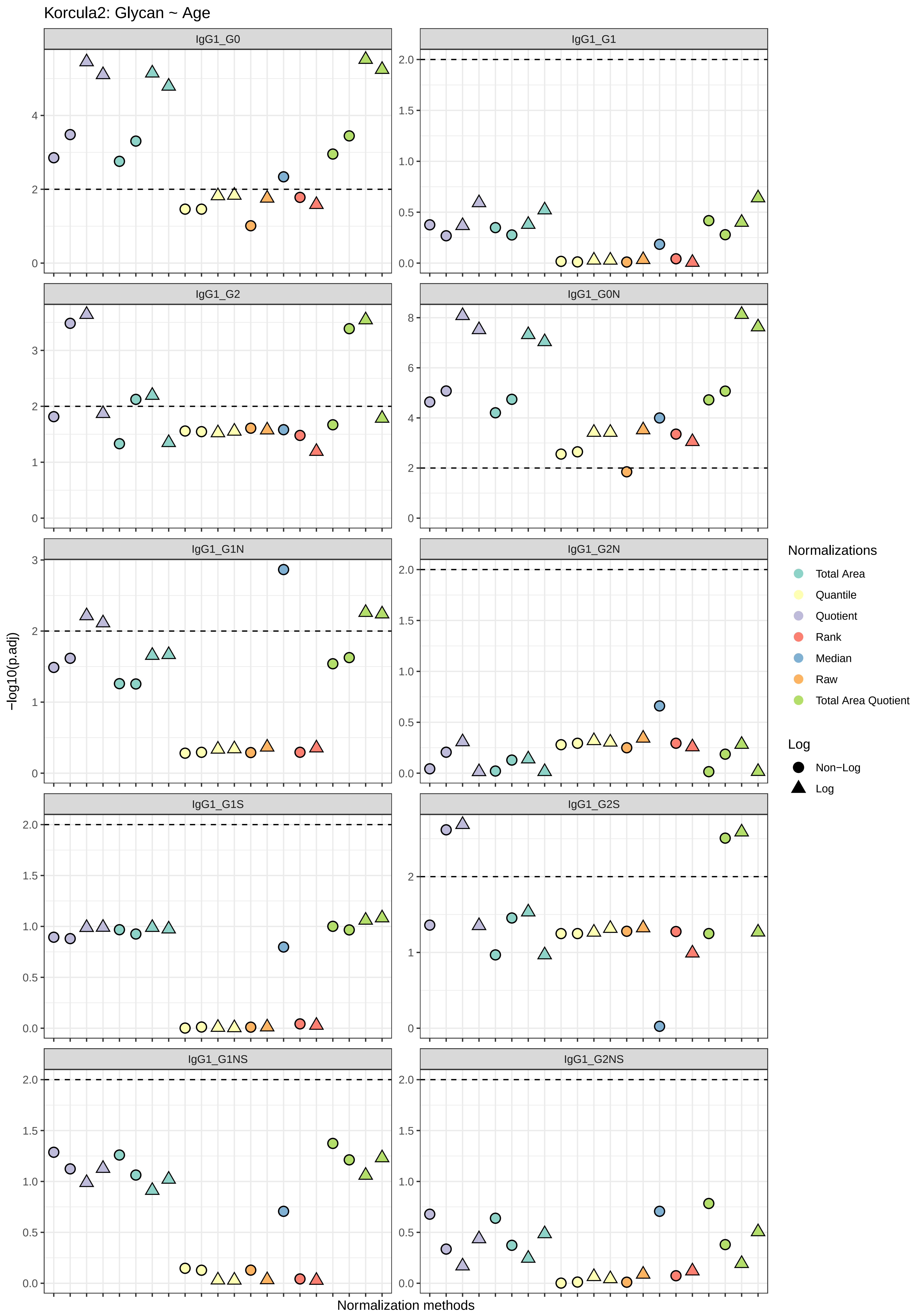

Korcula2: Glycan ~ Age
IgG1_G0
IgG1_G1
IgG1_G2
IgG1_G0N
IgG1_G1N
IgG1_G2N
IgG1_G1S
IgG1_G2S
IgG1_G1NS
IgG1_G2NS
−log10(p.adj)
Normalization methods
Normalizations
Total Area
Quantile
Quotient
Rank
Median
Raw
Total Area Quotient
Log
Non−Log
Log

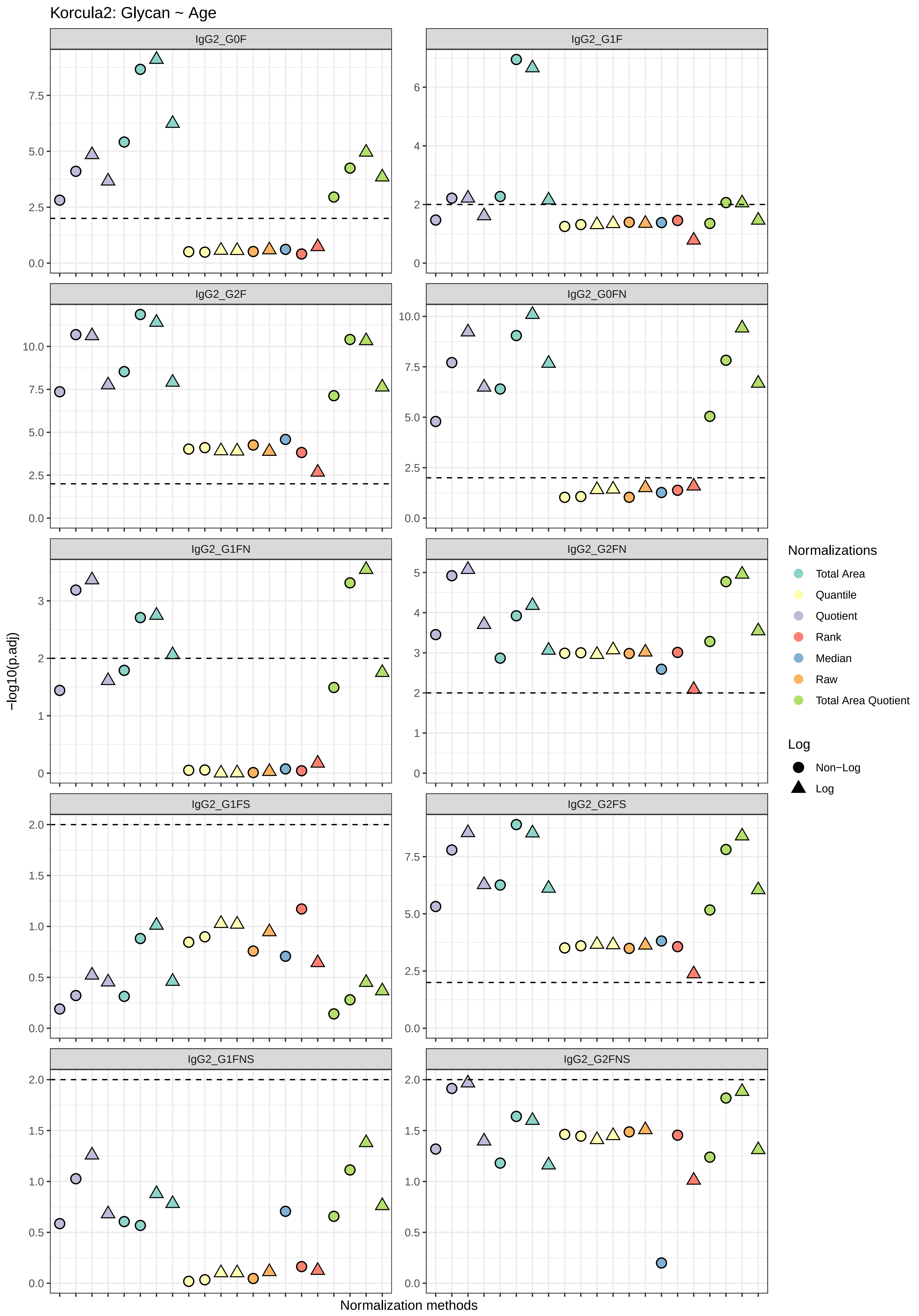

Korcula2: Glycan ~ Age
IgG2_G0F
IgG2_G1F
IgG2_G2F
IgG2_G0FN
IgG2_G1FN
IgG2_G2FN
IgG2_G1FS
IgG2_G2FS
IgG2_G1FNS
IgG2_G2FNS
−log10(p.adj)
Normalization methods
Normalizations
Total Area
Quantile
Quotient
Rank
Median
Raw
Total Area Quotient
Log
Non−Log
Log

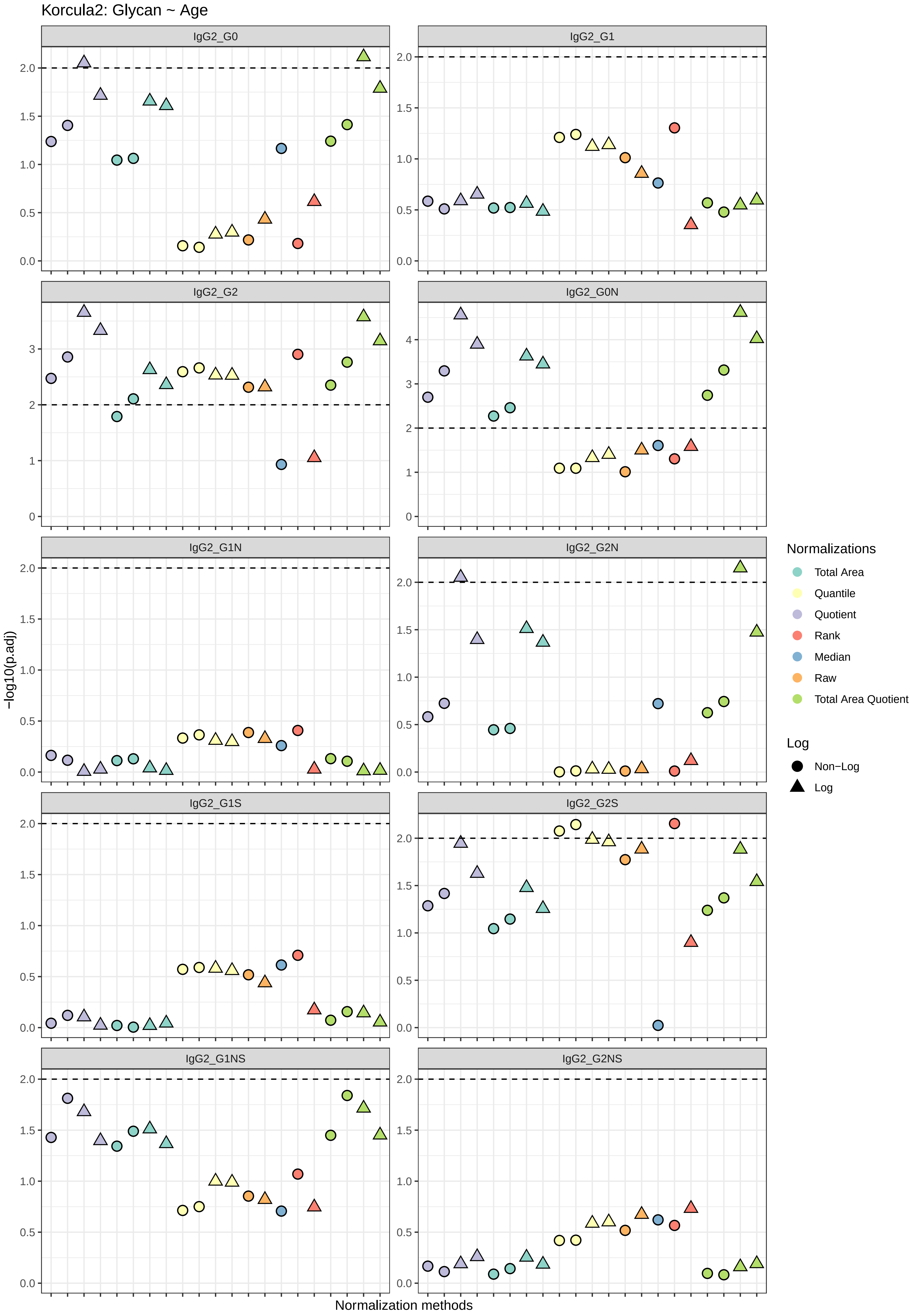

Korcula2: Glycan ~ Age
IgG2_G0
IgG2_G1
IgG2_G2
IgG2_G0N
IgG2_G1N
IgG2_G2N
IgG2_G1S
IgG2_G2S
IgG2_G1NS
IgG2_G2NS
−log10(p.adj)
Normalization methods
Normalizations
Total Area
Quantile
Quotient
Rank
Median
Raw
Total Area Quotient
Log
Non−Log
Log

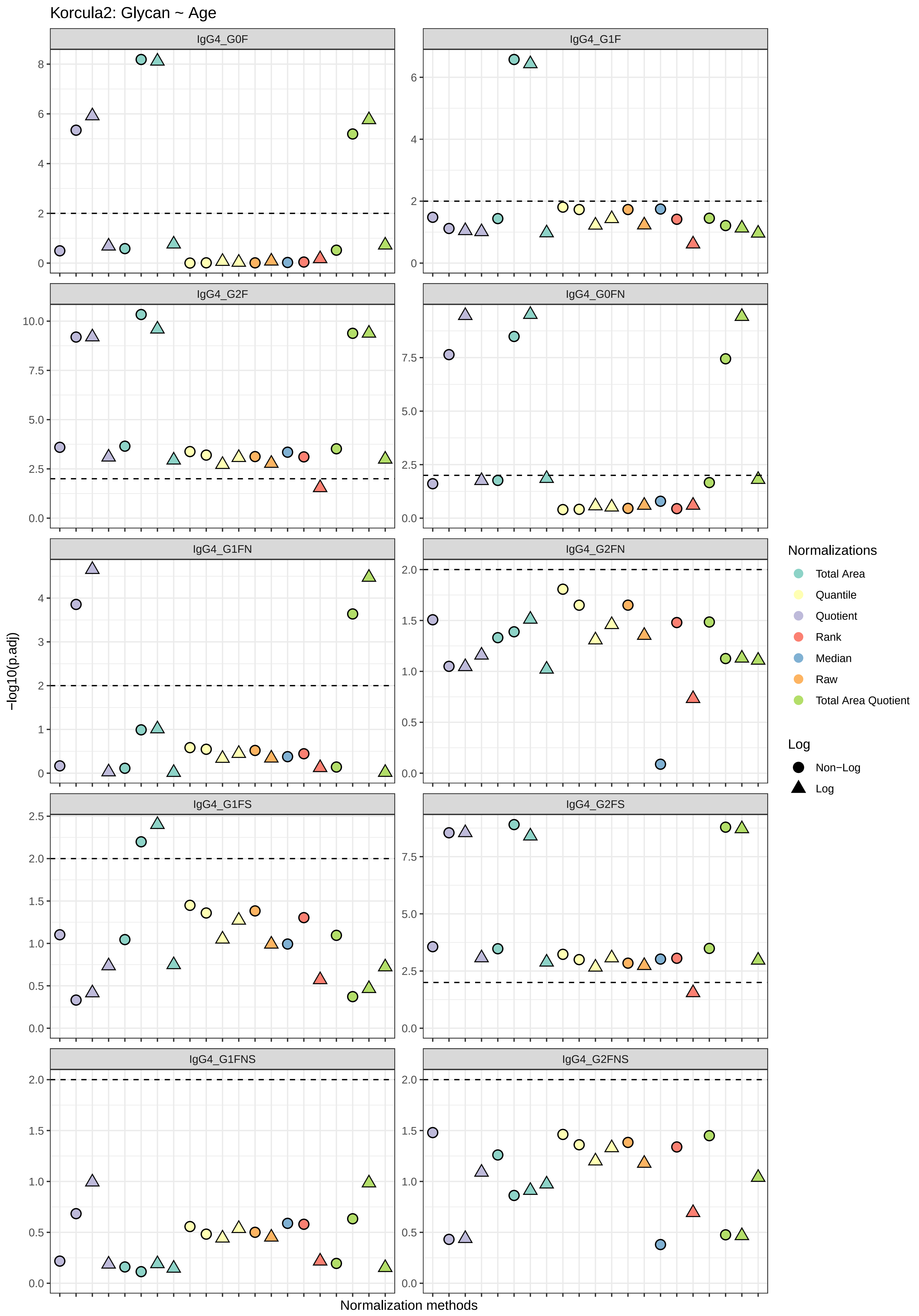
Korcula2: Glycan ~ Age
IgG4_G0F
IgG4_G1F
IgG4_G2F
IgG4_G0FN
IgG4_G1FN
IgG4_G2FN
IgG4_G1FS
IgG4_G2FS
IgG4_G1FNS
IgG4_G2FNS
−log10(p.adj)
Normalization methods
Normalizations
Total Area
Quantile
Quotient
Rank
Median
Raw
Total Area Quotient
Log
Non−Log
Log

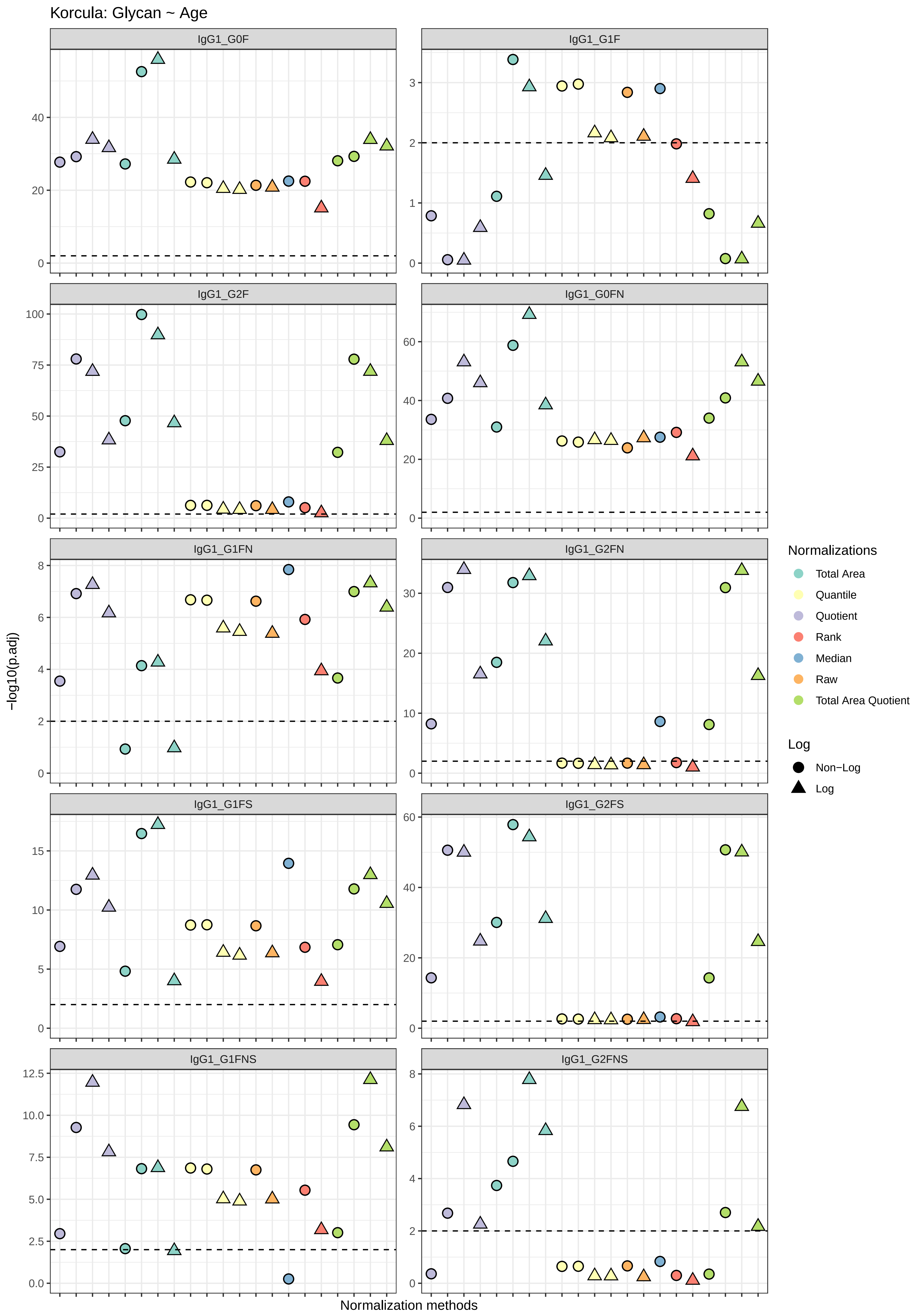

Korcula: Glycan ~ Age
IgG1_G0F
IgG1_G1F
IgG1_G2F
IgG1_G0FN
IgG1_G1FN
IgG1_G2FN
IgG1_G1FS
IgG1_G2FS
IgG1_G1FNS
IgG1_G2FNS
−log10(p.adj)
Normalization methods
Normalizations
Total Area
Quantile
Quotient
Rank
Median
Raw
Total Area Quotient
Log
Non−Log
Log

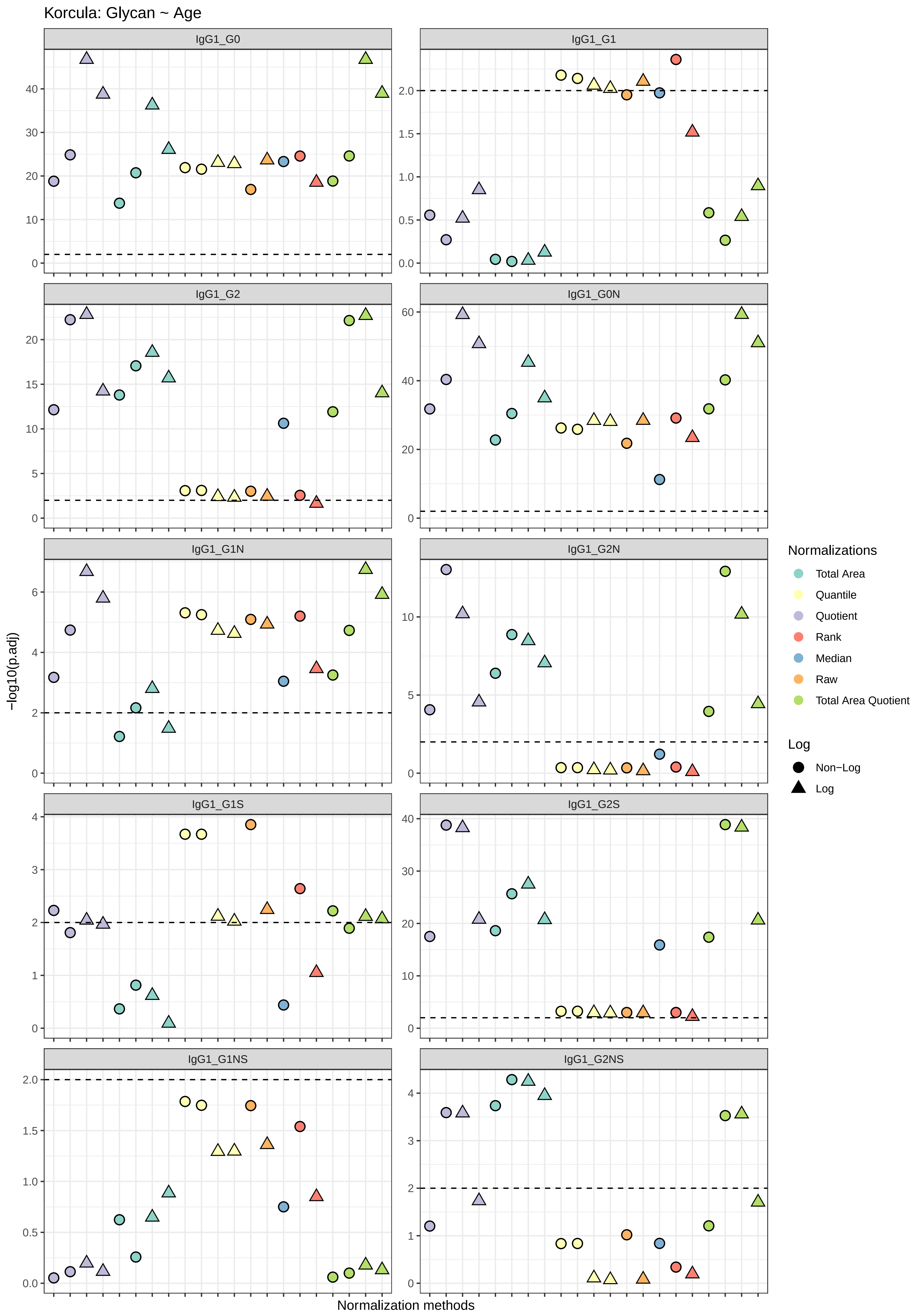
Korcula: Glycan ~ Age
IgG1_G0
IgG1_G1
IgG1_G2
IgG1_G0N
IgG1_G1N
IgG1_G2N
IgG1_G1S
IgG1_G2S
IgG1_G1NS
IgG1_G2NS
−log10(p.adj)
Normalization methods
Normalizations
Total Area
Quantile
Quotient
Rank
Median
Raw
Total Area Quotient
Log
Non−Log
Log

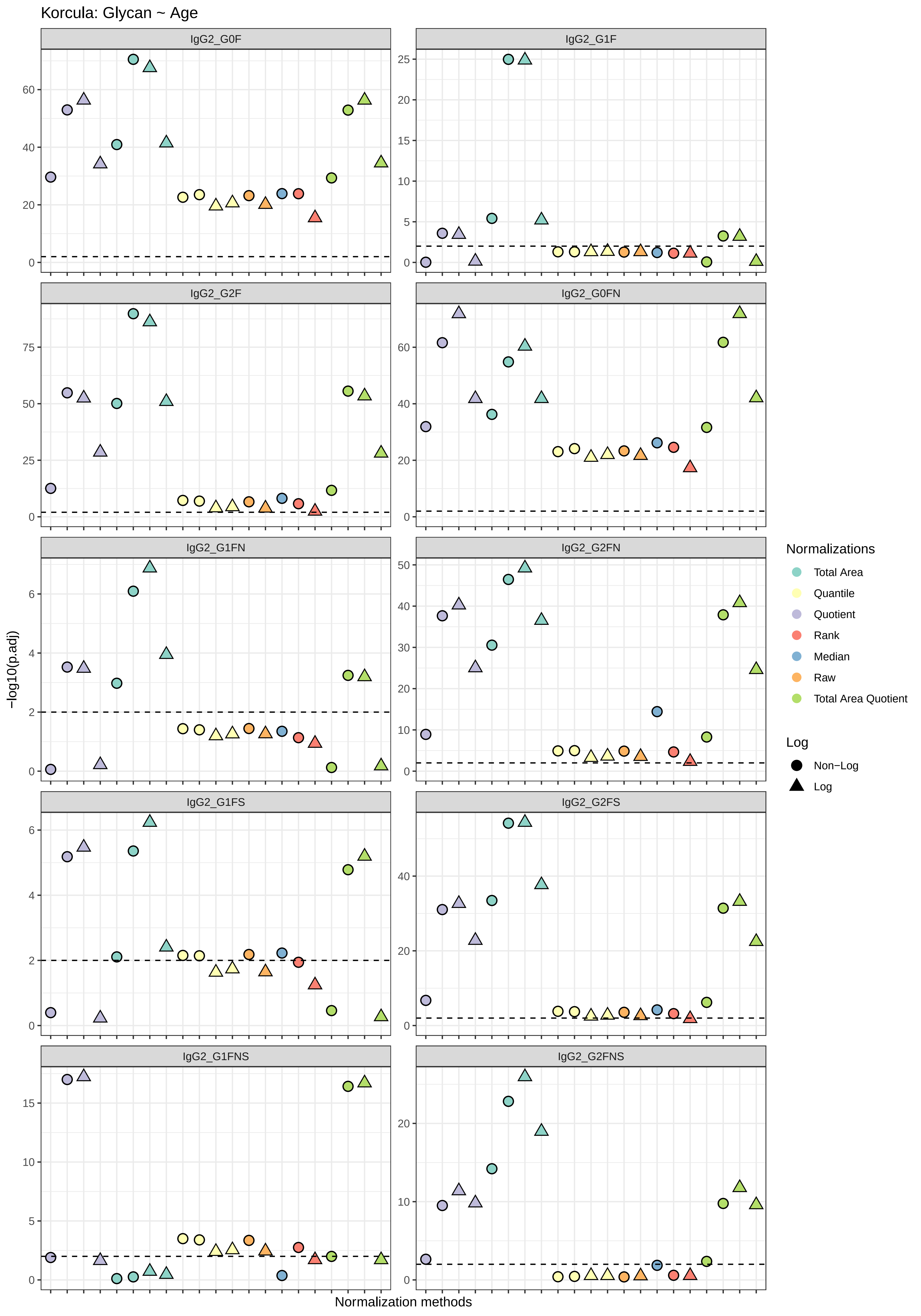

Korcula: Glycan ~ Age
IgG2_G0F
IgG2_G1F
IgG2_G2F
IgG2_G0FN
IgG2_G1FN
IgG2_G2FN
IgG2_G1FS
IgG2_G2FS
IgG2_G1FNS
IgG2_G2FNS
−log10(p.adj)
Normalization methods
Normalizations
Total Area
Quantile
Quotient
Rank
Median
Raw
Total Area Quotient
Log
Non−Log
Log

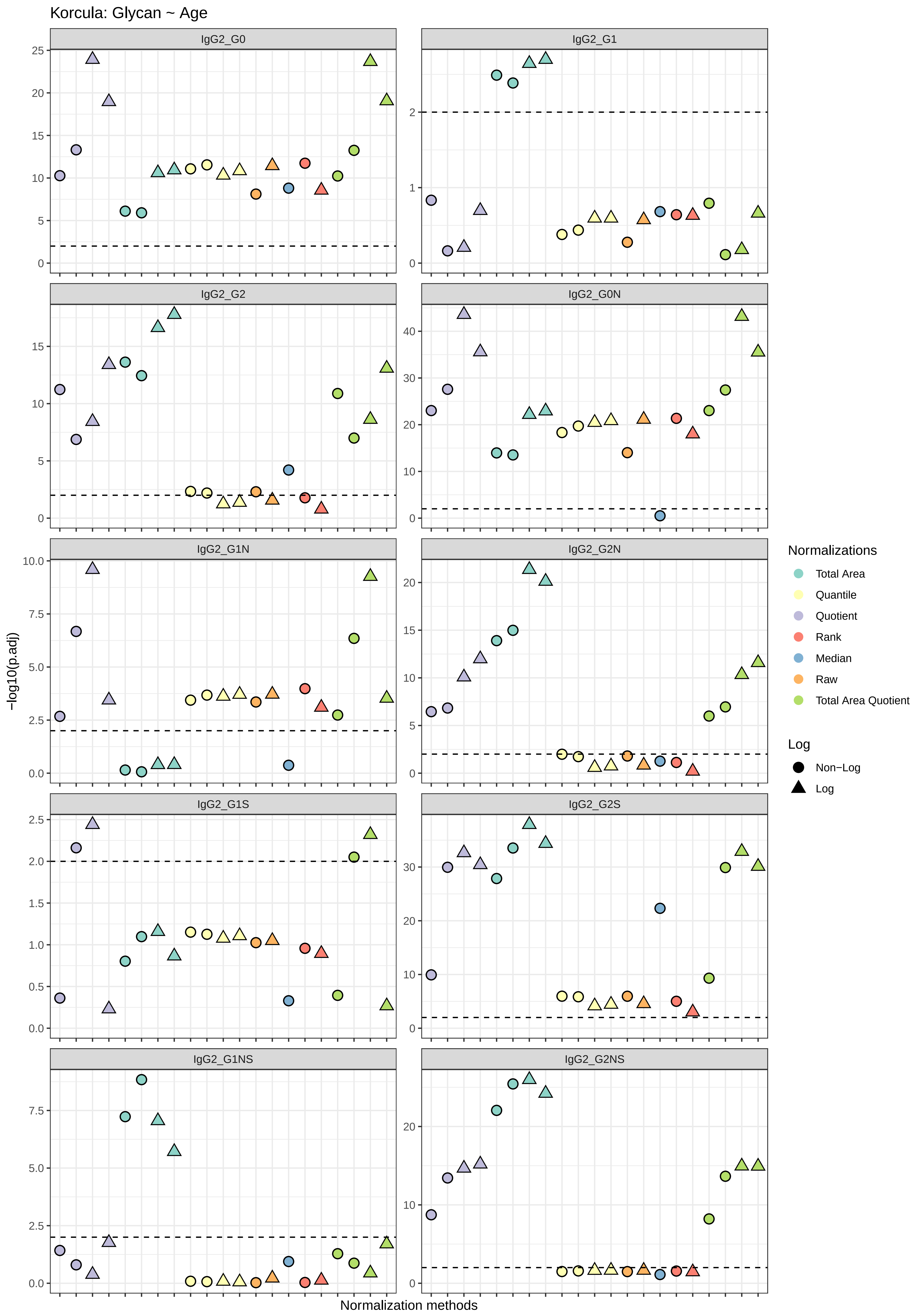

Korcula: Glycan ~ Age
IgG2_G0
IgG2_G1
IgG2_G2
IgG2_G0N
IgG2_G1N
IgG2_G2N
IgG2_G1S
IgG2_G2S
IgG2_G1NS
IgG2_G2NS
−log10(p.adj)
Normalization methods
Normalizations
Total Area
Quantile
Quotient
Rank
Median
Raw
Total Area Quotient
Log
Non−Log
Log

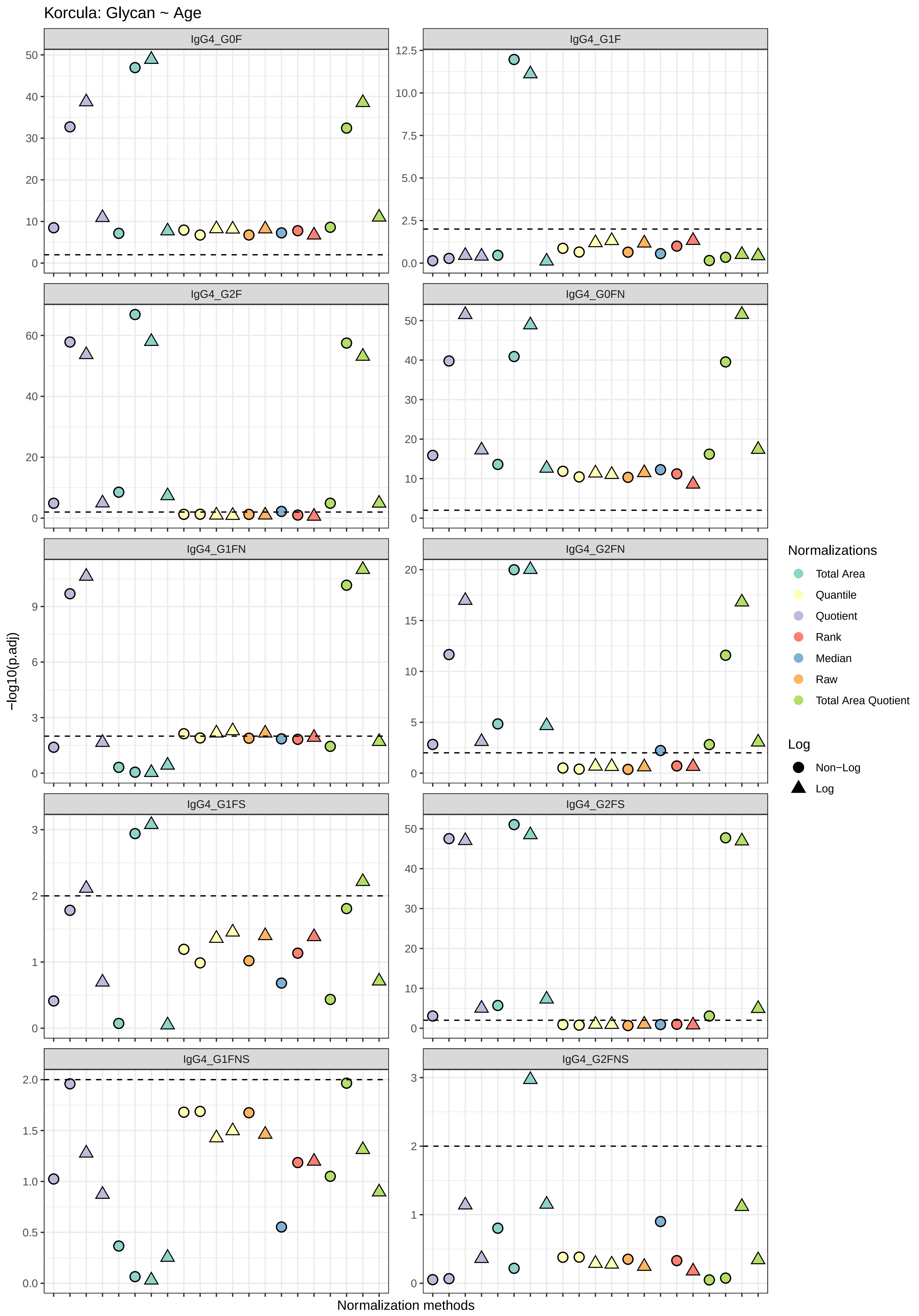

Korcula: Glycan ~ Age
IgG4_G0F
IgG4_G1F
IgG4_G2F
IgG4_G0FN
IgG4_G1FN
IgG4_G2FN
IgG4_G1FS
IgG4_G2FS
IgG4_G1FNS
IgG4_G2FNS
−log10(p.adj)
Normalization methods
Normalizations
Total Area
Quantile
Quotient
Rank
Median
Raw
Total Area Quotient
Log
Non−Log
Log

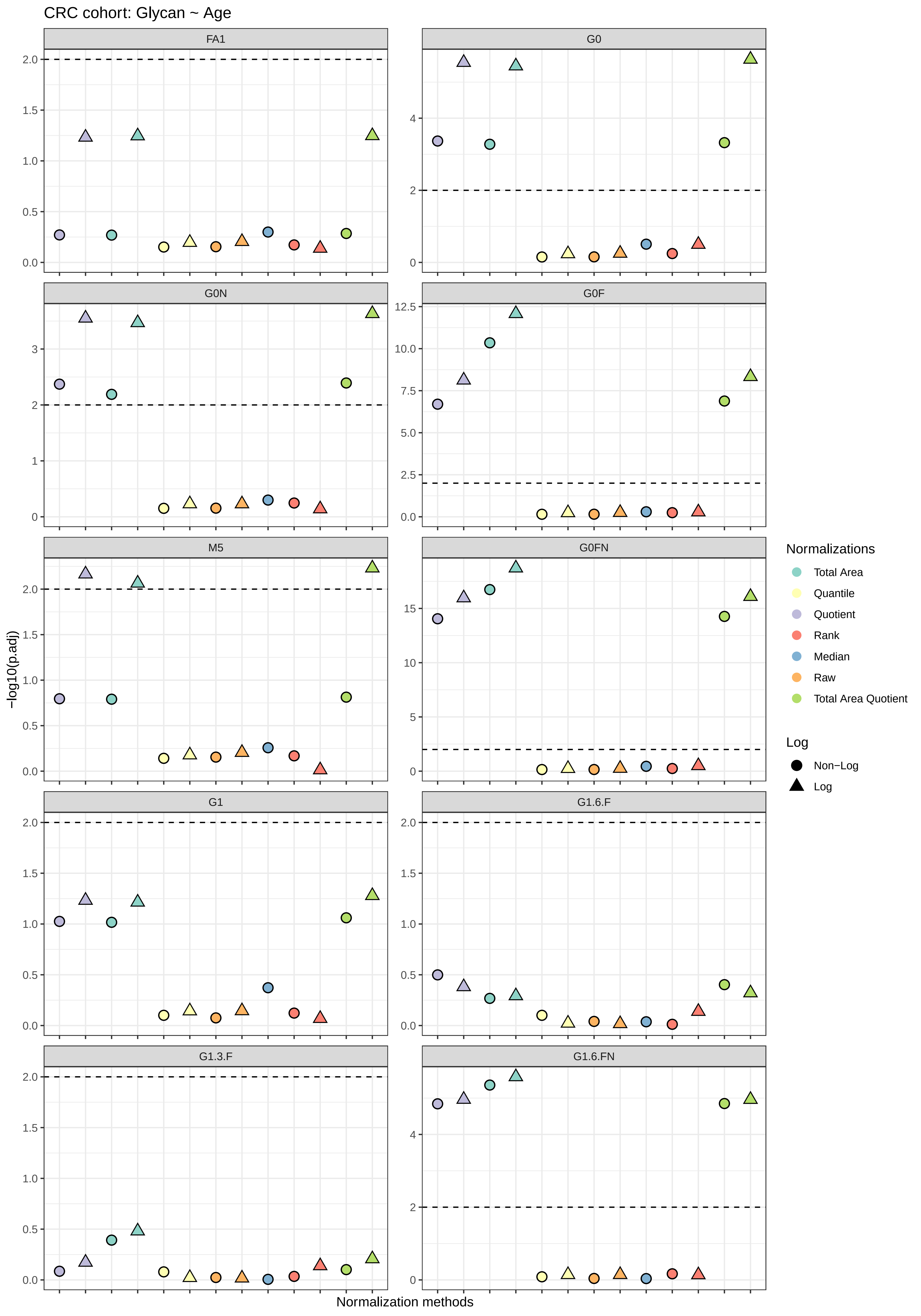

CRC cohort: Glycan ~ Age
FA1
G0
G0N
G0F
M5
G0FN
G1
G1.6.F
G1.3.F
G1.6.FN
−log10(p.adj)
Normalization methods
Normalizations
Total Area
Quantile
Quotient
Rank
Median
Raw
Total Area Quotient
Log
Non−Log
Log

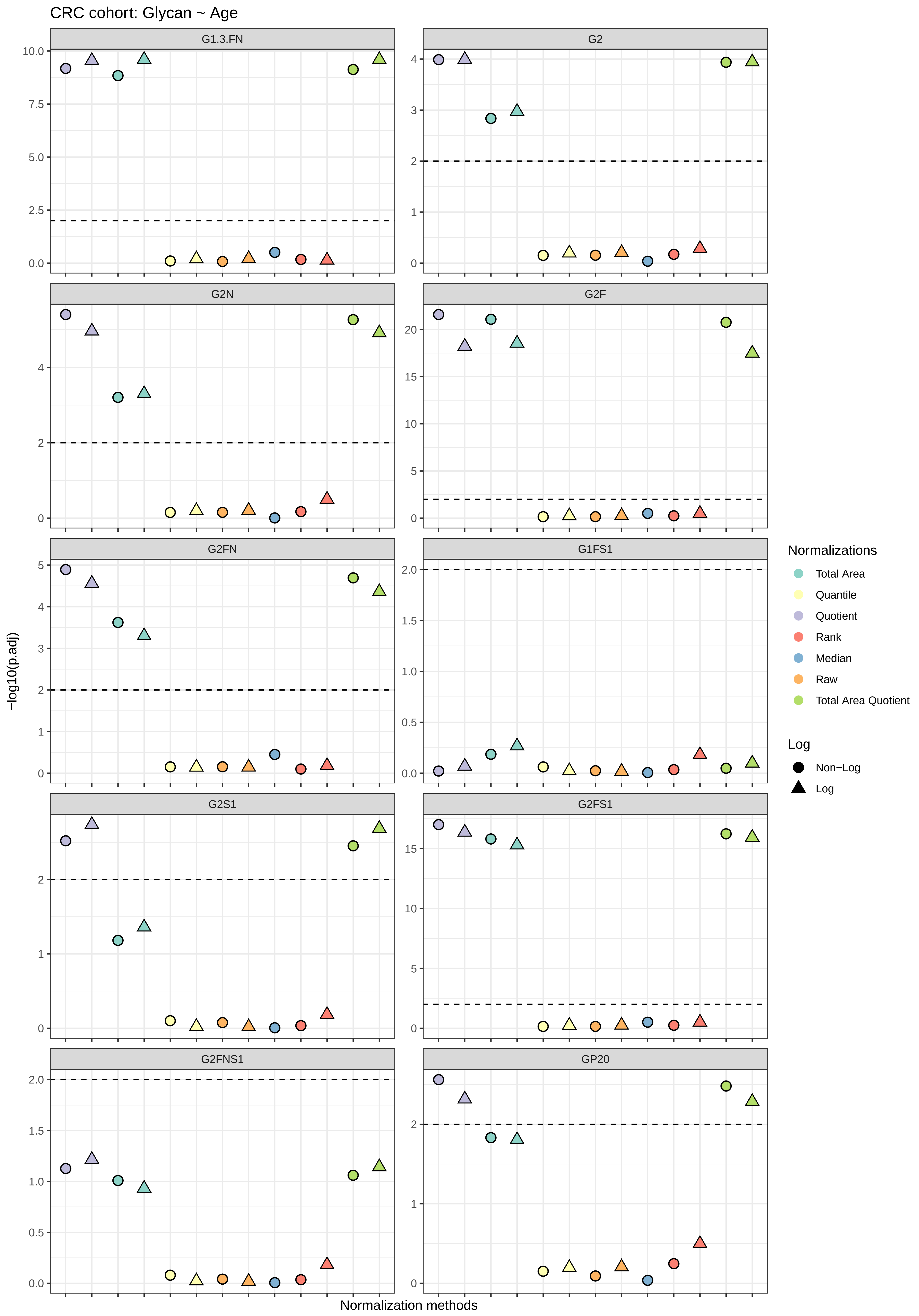

CRC cohort: Glycan ~ Age
G1.3.FN
G2
G2N
G2F
G2FN
G1FS1
G2S1
G2FS1
G2FNS1
GP20
−log10(p.adj)
Normalization methods
Normalizations
Total Area
Quantile
Quotient
Rank
Median
Raw
Total Area Quotient
Log
Non−Log
Log

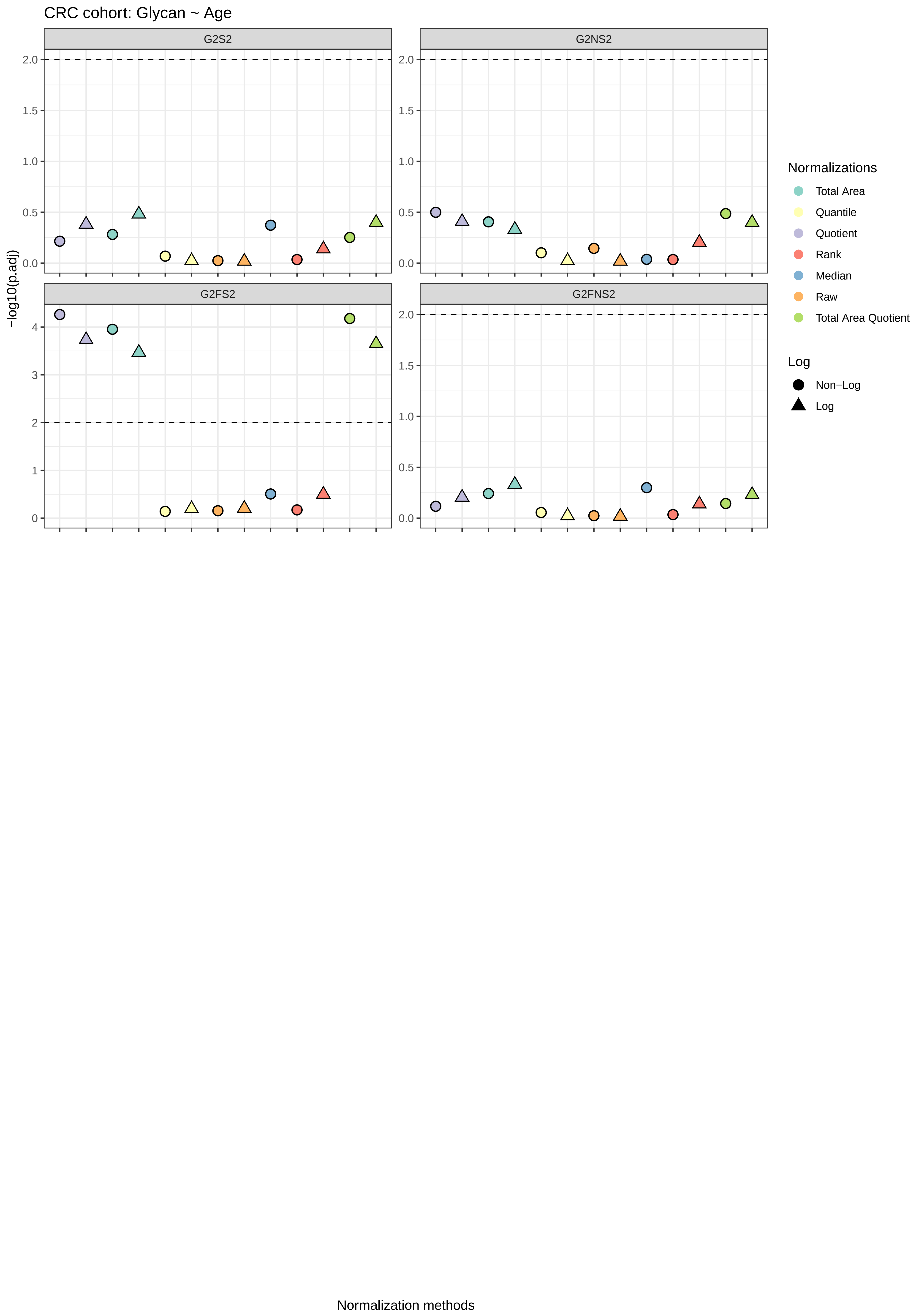

CRC cohort: Glycan ~ Age
G2S2
G2NS2
G2FS2
G2FNS2
−log10(p.adj)
Normalization methods
Normalizations
Total Area
Quantile
Quotient
Rank
Median
Raw
Total Area Quotient
Log
Non−Log
Log

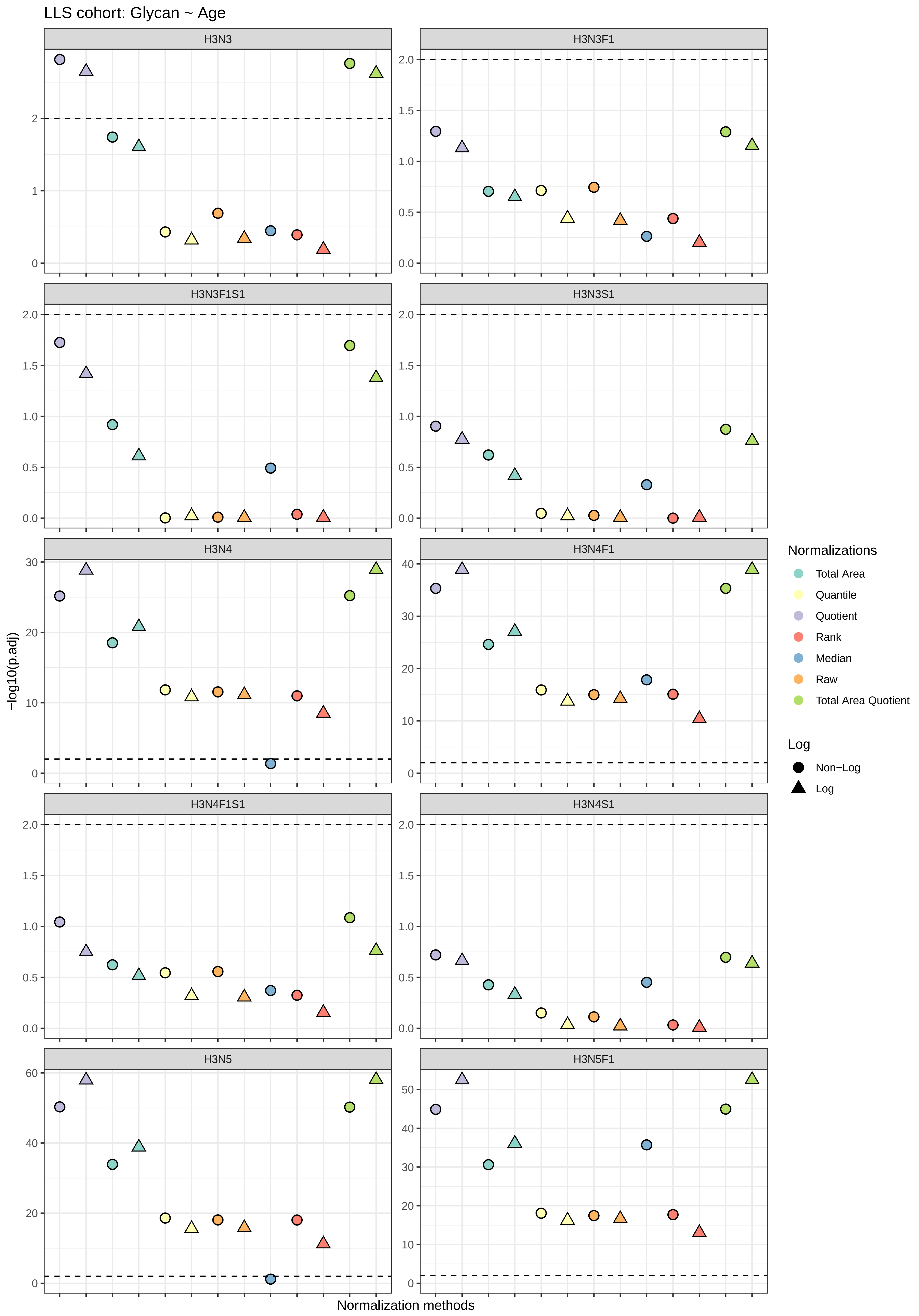
LLS cohort: Glycan ~ Age
H3N3
H3N3F1
H3N3F1S1
H3N3S1
H3N4
H3N4F1
H3N4F1S1
H3N4S1
H3N5
H3N5F1
−log10(p.adj)
Normalization methods
Normalizations
Total Area
Quantile
Quotient
Rank
Median
Raw
Total Area Quotient
Log
Non−Log
Log

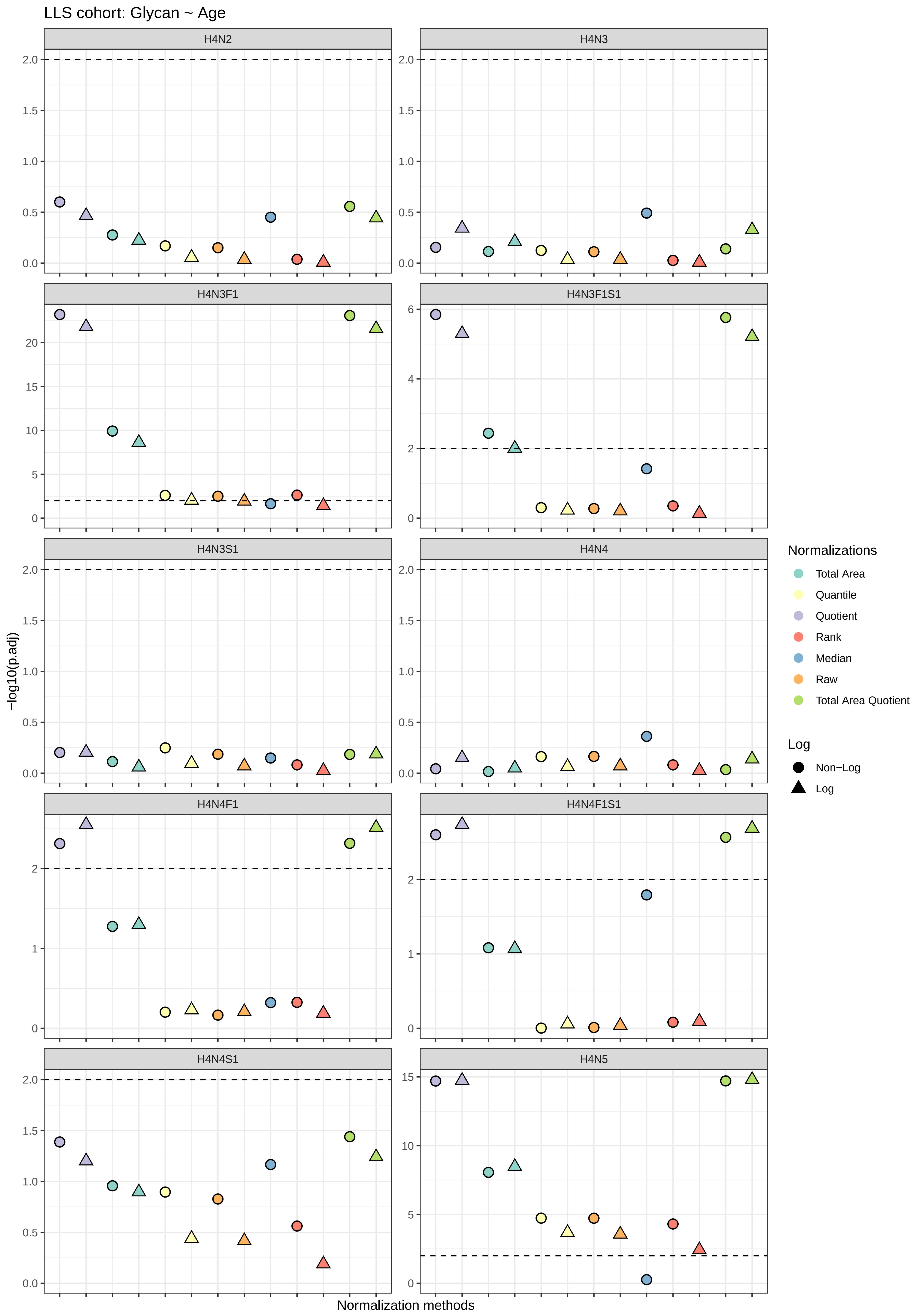
LLS cohort: Glycan ~ Age
H4N2
H4N3
H4N3F1
H4N3F1S1
H4N3S1
H4N4
H4N4F1
H4N4F1S1
H4N4S1
H4N5
−log10(p.adj)
Normalization methods
Normalizations
Total Area
Quantile
Quotient
Rank
Median
Raw
Total Area Quotient
Log
Non−Log
Log

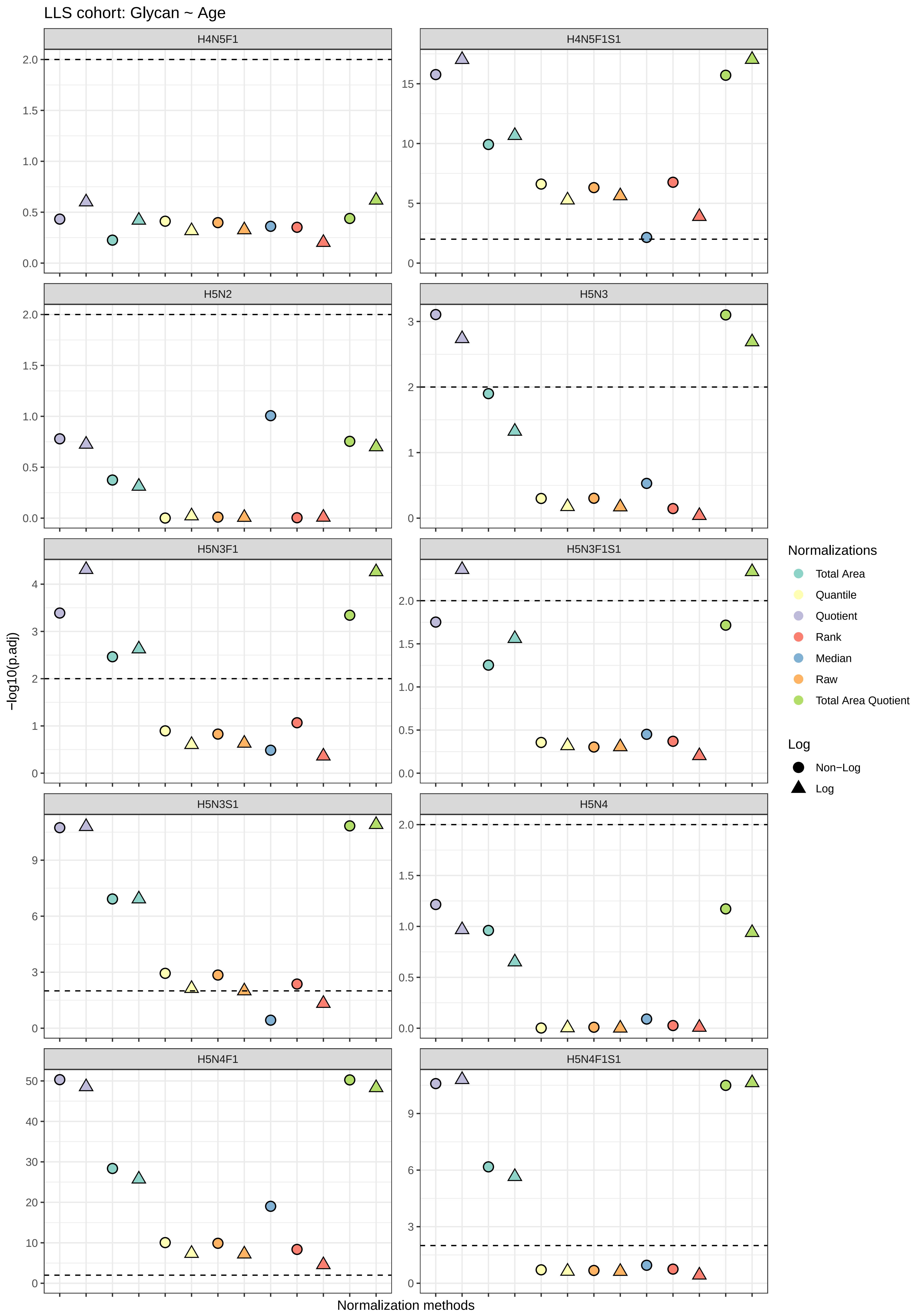

LLS cohort: Glycan ~ Age
H4N5F1
H4N5F1S1
H5N2
H5N3
H5N3F1
H5N3F1S1
H5N3S1
H5N4
H5N4F1
H5N4F1S1
−log10(p.adj)
Normalization methods
Normalizations
Total Area
Quantile
Quotient
Rank
Median
Raw
Total Area Quotient
Log
Non−Log
Log

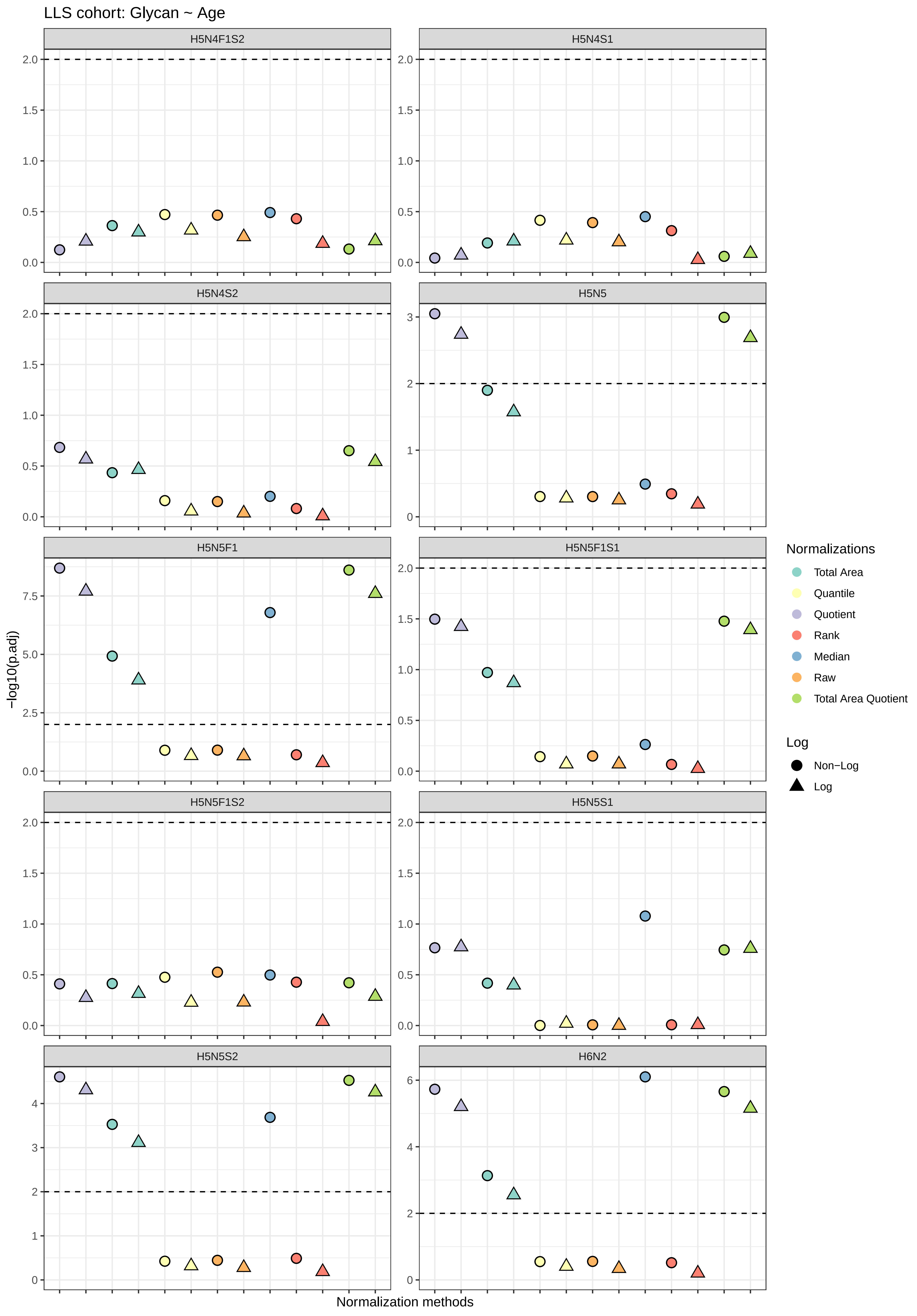

LLS cohort: Glycan ~ Age
H5N4F1S2
H5N4S1
H5N4S2
H5N5
H5N5F1
H5N5F1S1
H5N5F1S2
H5N5S1
H5N5S2
H6N2
−log10(p.adj)
Normalization methods
Normalizations
Total Area
Quantile
Quotient
Rank
Median
Raw
Total Area Quotient
Log
Non−Log
Log

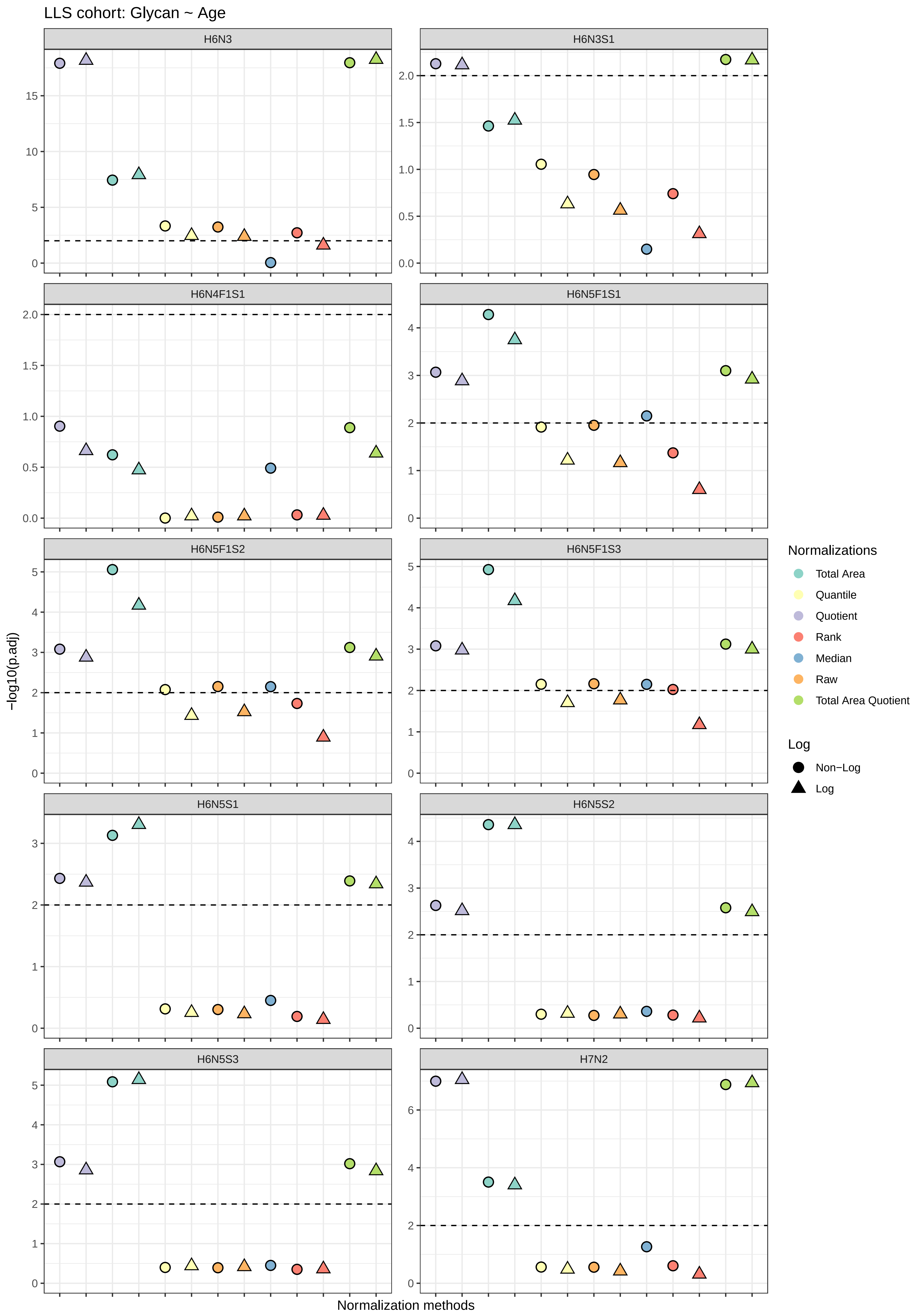

LLS cohort: Glycan ~ Age
H6N3
H6N3S1
H6N4F1S1
H6N5F1S1
H6N5F1S2
H6N5F1S3
H6N5S1
H6N5S2
H6N5S3
H7N2
−log10(p.adj)
Normalization methods
Normalizations
Total Area
Quantile
Quotient
Rank
Median
Raw
Total Area Quotient
Log
Non−Log
Log

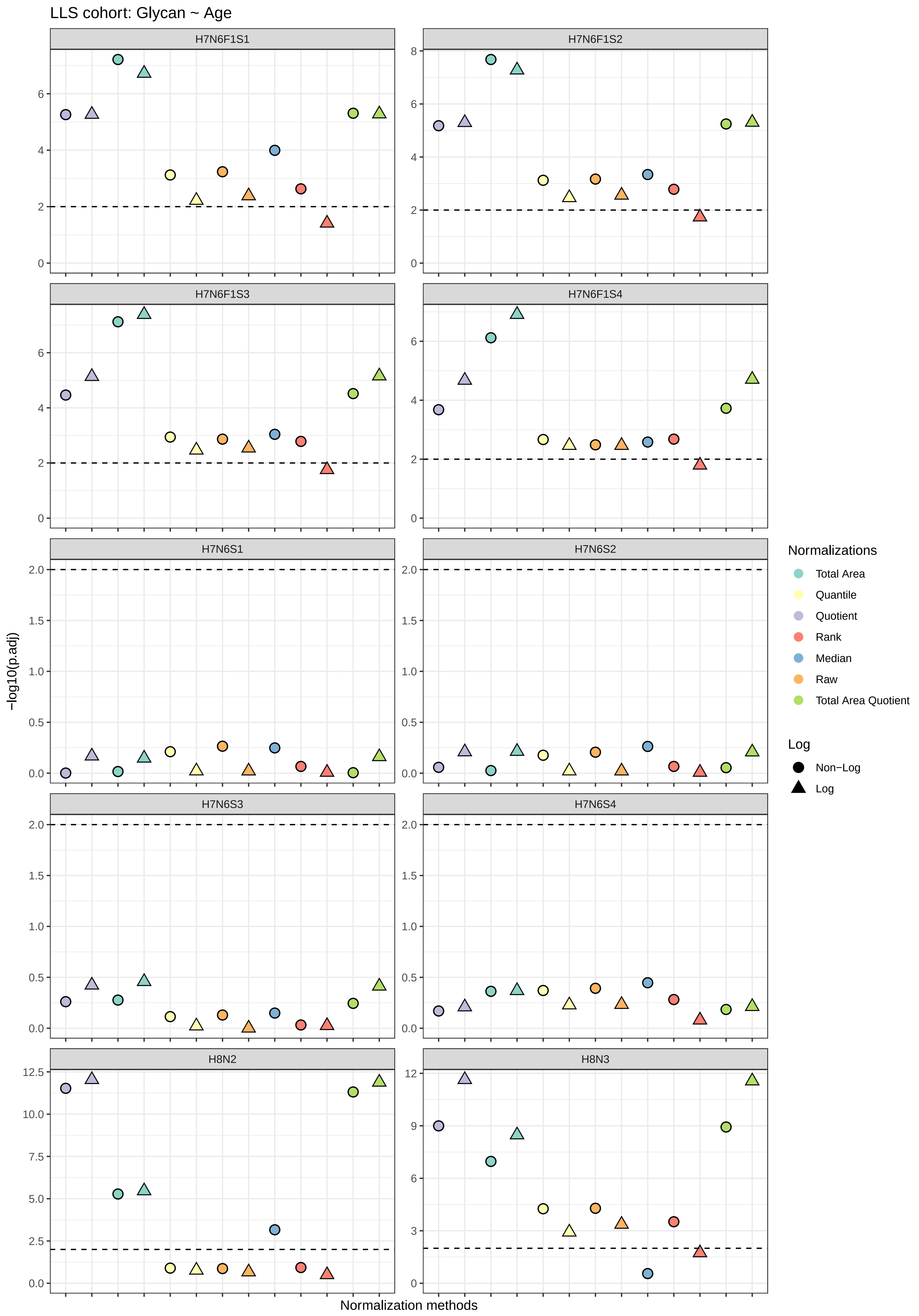
LLS cohort: Glycan ~ Age
H7N6F1S1
H7N6F1S2
H7N6F1S3
H7N6F1S4
H7N6S1
H7N6S2
H7N6S3
H7N6S4
H8N2
H8N3
−log10(p.adj)
Normalization methods
Normalizations
Total Area
Quantile
Quotient
Rank
Median
Raw
Total Area Quotient
Log
Non−Log
Log

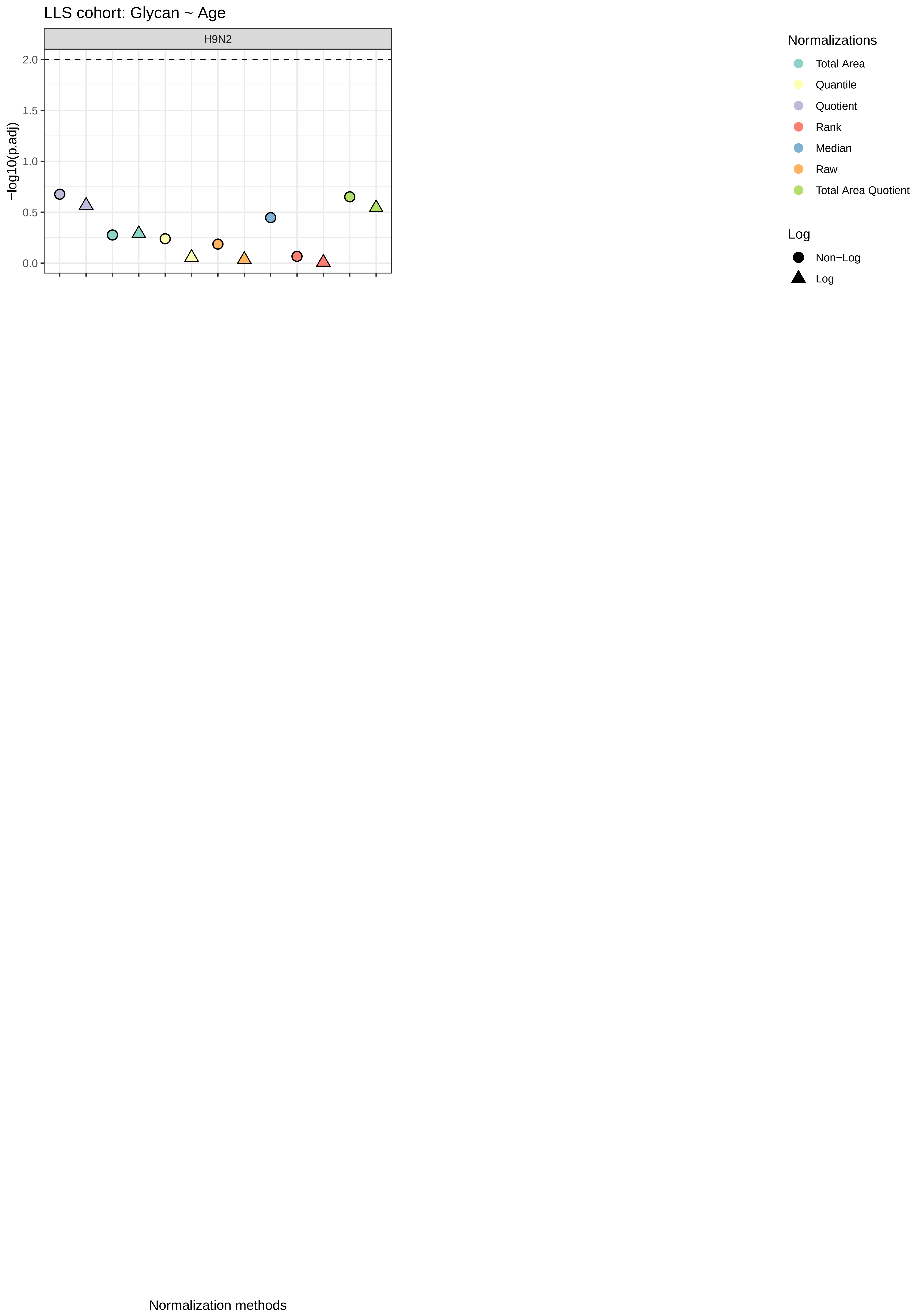
LLS cohort: Glycan ~ Age
H9N2
−log10(p.adj)
2.0
1.5
1.0
0.5
0.0
Normalization methods
Normalizations
Total Area
Quantile
Quotient
Rank
Median
Raw
Total Area Quotient
Log
Non−Log
Log
